# Glomerular expression pattern of long non-coding RNAs in the type 2 diabetes mellitus BTBR mouse model

**DOI:** 10.1038/s41598-019-46180-1

**Published:** 2019-07-05

**Authors:** Simone Reichelt-Wurm, Tobias Wirtz, Dominik Chittka, Maja Lindenmeyer, Robert M. Reichelt, Sebastian Beck, Panagiotis Politis, Aristidis Charonis, Markus Kretz, Tobias B. Huber, Shuya Liu, Bernhard Banas, Miriam C. Banas

**Affiliations:** 1 Department of Nephrology, University Hospital Regensburg, Regensburg, Germany; 2 Nephrological Center, Medical Clinic and Policlinic IV, University Hospital of Munich, Munich, Germany; 3 III. Department of Medicine, University Medical Center Hamburg-Eppendorf, Hamburg, Germany; 4 Department of Biochemistry, Genetics and Microbiology, Institute of Microbiology, University of Regensburg, Regensburg, Germany; 5 Center for Basic Research, Biomedical Research Foundation of the Academy of Athens, Athens, Greece; 6 Center for Clinical, Experimental Surgery and Translational Research, Biomedical Research Foundation of the Academy of Athens, Athens, Greece; 7 Institute of Biochemistry, Genetics and Microbiology, University of Regensburg, Regensburg, Germany

**Keywords:** Chronic kidney disease, RNA, Microarrays, Type 2 diabetes

## Abstract

The prevalence of type 2 diabetes mellitus (T2DM) and by association diabetic nephropathy (DN) will continuously increase in the next decades. Nevertheless, the underlying molecular mechanisms are largely unknown and studies on the role of new actors like long non-coding RNAs (lncRNAs) barely exist. In the present study, the inherently insulin-resistant mouse strain “black and tan, brachyuric” (BTBR) served as T2DM model. While wild-type mice do not exhibit pathological changes, leptin-deficient diabetic animals develop a severe T2DM accompanied by a DN, which closely resembles the human phenotype. We analyzed the glomerular expression of lncRNAs from wild-type and diabetic BTBR mice (four, eight, 16, and 24 weeks) applying the “GeneChip Mouse Whole Transcriptome 1.0 ST” array. This microarray covered more lncRNA gene loci than any other array before. Over the observed time, our data revealed differential expression patterns of 1746 lncRNAs, which markedly differed from mRNAs. We identified protein-coding and non-coding genes, that were not only co-located but also co-expressed, indicating a potentially cis-acting function of these lncRNAs. *In vitro*-experiments strongly suggested a cell-specific expression of these lncRNA-mRNA-pairs. Additionally, protein-coding genes, being associated with significantly regulated lncRNAs, were enriched in various biological processes and pathways, that were strongly linked to diabetes.

## Introduction

Recent studies elucidated that more than 90% of the human genome is expressed as primary transcript^1^. However, only approximately 2% of all transcripts encode proteins while non-coding transcripts comprise the vast majority^2^.

Long non-coding RNAs (lncRNAs) represent the largest group of non-coding RNAs (ncRNAs)^3^. They are characterized by their length (>200 nucleotides) and their absent protein-coding potential^4^. Furthermore, they share features with mRNAs like 5′-capping, 3′-polyadenylation, and splicing^5^. Depending on their genomic localization, lncRNAs can be classified as intragenic or intergenic. The latter, also referred as long intergenic ncRNAs (lincRNAs), localize within the genomic interval between two genes. Intragenic lncRNAs overlap with protein-coding genes on the same or opposite DNA strand and can have a sense or antisense orientation^3^.

LncRNAs can virtually influence every step of gene expression in an activating as well as repressing manner. This includes e. g. chromatin modification, transcriptional control, or mRNA stability^6^. For instance, the lncRNA metastasis associated lung adenocarcinoma transcript 1 (*MALAT1*) affects alternative splicing events^7^.

Since lncRNAs are involved in so many regulatory mechanisms, they represent key players in various cancers^8,9^ and other diseases^10,11^. In the renal context, lncRNAs affect acute kidney injury^12^, fibrosis, and inflammation^13,14^. Recently, growing evidence emerged that lncRNAs are not only highly relevant in the pathogenesis of diabetes in general^15^, but also in diabetic nephropathy (DN) in particular^16–18^.

According to the International Diabetes Federation^19^, 415 million people worldwide suffered from diabetes in 2015. By 2040, the global diabetes prevalence will further increase to ca. 642 million patients. Thereby, the vast majority are type 2 diabetics^19^. Affecting 42% of all diabetic patients, DN represents a significant microvascular complication as well as the leading cause for end-stage renal disease^20^. DN is associated with albuminuria and a steadily decreasing glomerular filtration rate. Histological manifestations include mesangial expansion, glomerulosclerosis, extracapillary hypercellularity, interstitial fibrosis, and inflammation^20–22^.

Despite the known risk factors and disease symptoms, the underlying mechanisms of DN remain largely unclear. Studies indicate that glomerular cells are primarily involved in the development of DN^23^. To investigate the molecular basis, we used the mouse strain “black and tan, brachyuric” (BTBR). This strain is inherently insulin-resistant. But while there are no pathological changes in wildtype (WT) BTBR mice, leptin-deficient BTBR obese/obese (ob/ob) mice develop a severe type 2 diabetes mellitus (T2DM), which is accompanied by a DN, that closely resembles the human phenotype^24^.

To elucidate the relevance of glomerular expressed genes in the development of DN, we conducted a microarray experiment based on glomerular RNA from BTBR WT and ob/ob mice. In our previous study, we already analyzed differentially expressed (coding) genes (DEGs)^25^. Here, we focused on differentially expressed lncRNAs, which were spatially associated with protein-coding genes; meaning the lncRNA encodes either within a transcriptional unit or in a distance less than 50 kb. Based on already existing data^26,27^, these lncRNAs might have a cis-acting function.

## Results

### Expression pattern of lncRNAs

**Figure 1:**
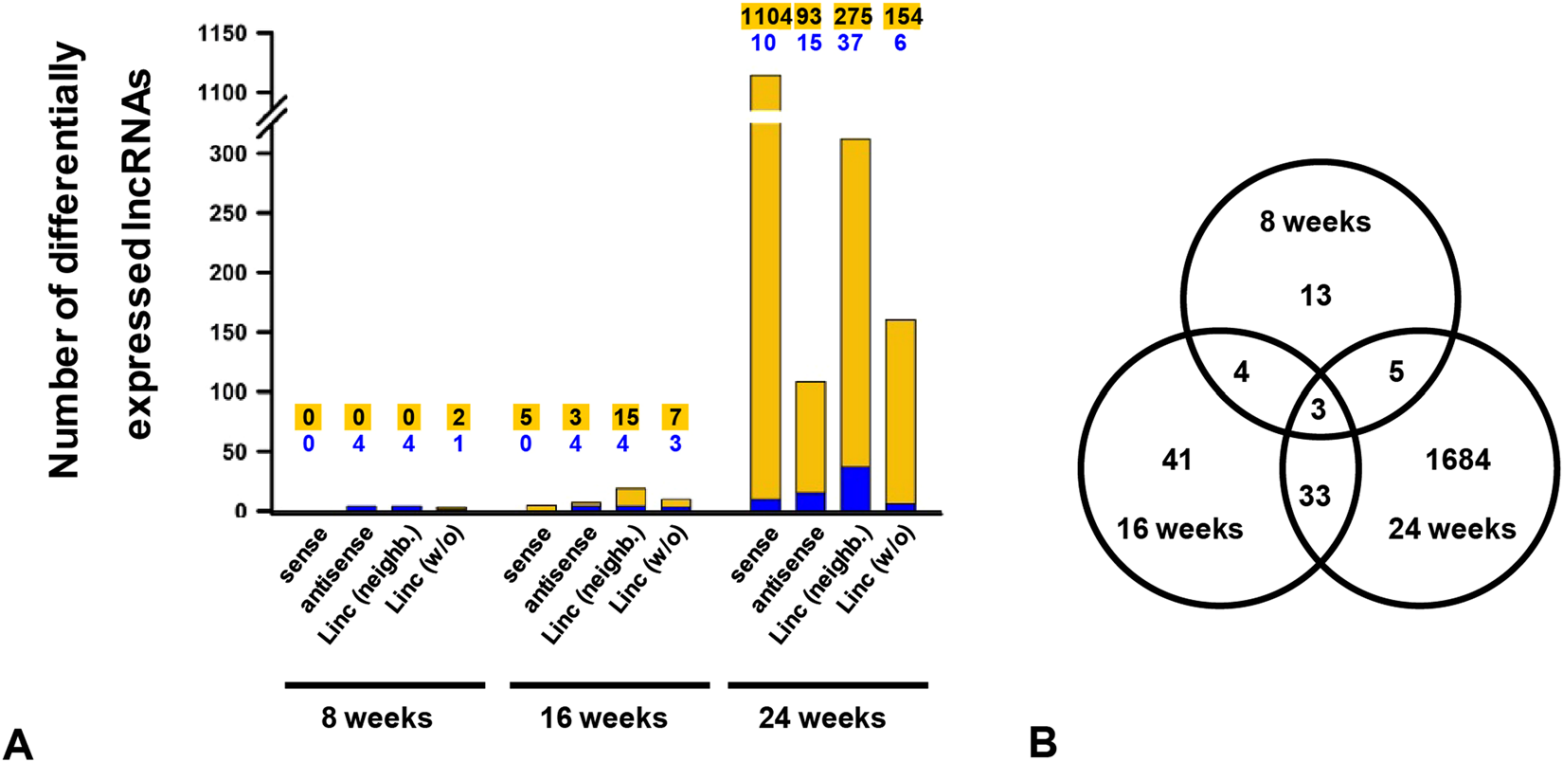
Expression of lncRNAs in BTBR ob/ob mice compared to age-matched WT mice. (**A**) Expression pattern of lncRNAs in 8, 16, and 24 weeks old BTBR ob/ob mice vs. BTBR WT mice categorized in sense and antisense lncRNAs, as well as lincRNAs with or without neighboring mRNA within a distance of 50 kb up- or downstream. The yellow part of each bar represents the number of upregulated transcripts and the blue part represents the number of downregulated transcripts. (**B**) The Venn diagram shows the total number of differentially expressed lncRNAs per age group.

For our analyses, BTBR WT and BTBR ob/ob mice from the age of four to 24 weeks were examined to illustrate the progression of glomerular damage in T2DM (Supplementary Fig. 1). Since glomerular transcripts are underrepresented in whole-kidney samples, we used RNA extracted from sieved glomeruli (Supplementary Fig. 2). We already demonstrated a correlation between the increasing severity of glomerular damage and the increasing number of DEGs^25^. Additionally, also many non-coding transcripts exhibited an altered expression pattern with a fold change above 2.0 or below 0.5, and a significant false discovery rate (FDR) p-value < 0.05. Since we wanted to focus on lncRNAs, other non-coding transcripts like ribosomal RNAs, small nuclear RNAs, or piwi-interacting RNAs were excluded. Comparably to mRNAs^25^, more lncRNAs were differentially expressed in older animals, having a pronounced diabetic phenotype (Fig. 1A). We ascertained eleven lncRNAs in eight weeks old mice, 41 lncRNAs in 16 weeks old mice, and 1694 lncRNAs in the oldest age group. In four weeks old mice, differentially expressed lncRNAs were not found. We categorized these lncRNAs according to their genomic localization as intergenic or intragenic. The latter were further characterized regarding their orientation as sense or antisense transcripts. Furthermore, we analyzed if lincRNAs were encoded up to 50 kb up- or downstream from a coding gene. In the eight weeks old group, only lincRNAs or lncRNAs in antisense orientation were detected. In addition, almost all lncRNAs were downregulated in eight weeks old age group. This pattern markedly changed over the time. Especially in 24 weeks old animals, the vast majority of differentially expressed lncRNAs exhibited a sense orientation and was upregulated. The Venn diagram in Fig. 1B gives further information about the distribution of lncRNAs in the three age groups. The Supplementary Table 1 specifies the transcripts being differentially expressed in two or three age groups.

### Co-localization of differentially expressed lncRNAs and mRNAs

In the following analysis, we wanted to identify differentially expressed lncRNAs, which are spatially associated with a DEGs (shown in our recent work^25^) (Table 1). LncRNAs were categorized in the same manner as described above. Additionally, we examined if associated transcripts had the same or a converse expression pattern; meaning both RNAs were upregulated or downregulated, respectively. Alternatively, one transcript was upregulated while the other was downregulated.

**Table 1:** Regulation pattern of co-located differentially expressed mRNAs and lncRNAs.

Regulation pattern	Age in weeks			
	4	8	16	24
**Regulation of differentially expressed genes and their corresponding sense transcripts**				
Protein coding gene ↑ Sense transcript ↑	0	0	1	48
Protein coding gene ↑ Sense transcript ↓	0	0	0	8
Protein coding gene ↓ Sense transcript ↓	0	0	0	4
Number of genes with corresponding sense transcript (n)	0	0	1	60
**Regulation of differentially expressed genes and their corresponding antisense transcripts**				
Protein coding gene ↑ Antisense transcript ↑	0	0	2	3
Protein coding gene ↑ Antisense transcript ↓	0	0	0	7
Protein coding gene ↓ Antisense transcript ↓	0	3	1	4
Number of genes with corresponding antisense transcript (n)	0	3	3	14
**Regulation of differentially expressed genes and their corresponding neighboring transcripts**				
Protein coding gene ↑ Neighboring transcript ↑	0	0	0	11
Protein coding gene ↑ Neighboring transcript ↓	0	0	0	13
Protein coding gene ↓ Neighboring transcript ↓	0	2	0	13
Number of genes with corresponding neighboring transcript (n)	0	2	0	37

Regulation pattern of differentially expressed mRNAs and their corresponding sense, antisense, or neighboring lncRNAs, sorted by the regulation patter and the age of mice in weeks. Arrows indicated an upregulation (↑) or downregulation (↓). Regarding sense and antisense transcripts, respectively, some mRNA-lncRNA-pairs were differentially expressed in more than one age group.

In all age groups, we found 76 lncRNAs, whose transcriptional unit overlapped with a DEG. Some DEGs harbored more than one lncRNA gene. Of these, 60 lncRNAs were transcribed in sense direction (Supplementary Table 2) and 16 lncRNAs in antisense direction (Supplementary Table 3). While lncRNAs in sense orientation often exhibited the same expression pattern like the spatial associated mRNA, such a coherence was not observed for antisense lncRNA-mRNA-pairs.

For lincRNAs, we also analyzed the genomic localization of a lincRNA and the adjacent mRNA. In total, we identified 39 differentially expressed lincRNAs being associated with a DEG. Thereby, 13 had a genomic distance less than 5 kb. Apparently, the genomic distance correlated with the expression pattern or the genomic locus. In 12 of 13 cases, the lincRNA and the mRNA exhibited the same expression pattern. Almost all lincRNAs localized downstream of the neighboring DEG (12 of 13) and eleven of 13 were encoded on the same DNA strand like the adjacent DEG.

### Genomic distribution of coding and non-coding genes

Next, we analyzed if there were salient patterns regarding the chromosomal distribution of genes. Information on the total number of coding and non-coding genes covered by this array was obtained from Affymetrix/Thermo Fisher Scientific (https://www.affymetrix.com/site/mainPage.affx).

**Figure 2:**
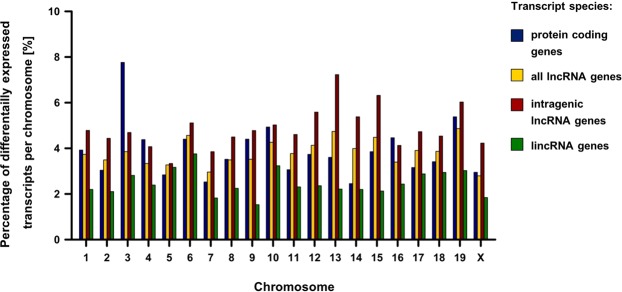
Percentage of differentially expressed mRNAs and lncRNAs per chromosome.

We calculated the ratio of differentially expressed mRNAs or lncRNAs relatively to the total number of encoded mRNAs or lncRNAs per chromosome. On average 3.89% (±1.21) of coding genes and 3.82% (±0.57) of lncRNA genes per chromosome were differentially expressed. Regarding the subsets of intragenic and intergenic lncRNA genes, respectively, 4.87% (±1.90) or 2.48% (±0.55) exhibited a differential expression (Fig. 2, Supplementary Fig. 3). Therefore, we could not identify a particular chromosome, which encoded significantly more or less coding or non-coding transcripts.

### Many coding genes encode more than one lncRNA gene within their transcriptional unit

In our study, 202 protein-coding genes harbored more than one lncRNA gene within their transcriptional unit. This list was led by the phosphodiesterase 4d (*Pde4d*, Supplementary Fig. 4) and the solute carrier family 8 (sodium/calcium exchanger) member 1 (*Slc8a1*). Their gene bodies contained twelve differentially expressed intragenic lncRNA genes. ADP-ribosylation factor-like 15 (*Arl15*) and adenosine kinase (*Adk*) harbored eleven or ten differentially expressed intragenic encoded lncRNA genes, respectively. In general, we observed markedly less differentially expressed antisense than sense lncRNAs. With four differentially expressed antisense lncRNAs, the insulin-like growth factor-2 receptor (*Igf2r*) harbored the most.

### Analysis and validation of expression changes

To validate our microarray results, we conducted quantitative polymerase chain reaction (qPCR) analyses. Pairs of DEGs and their corresponding also differentially expressed sense, antisense, or neighboring lncRNA genes were chosen (altogether 16 genes). Thereby, we wanted to focus on genes, that are relevant for diabetes or diabetic nephropathy. The following comparisons referred to the 24 weeks group; however, we also characterized the gene expression in the course of time.

**Figure 3:**
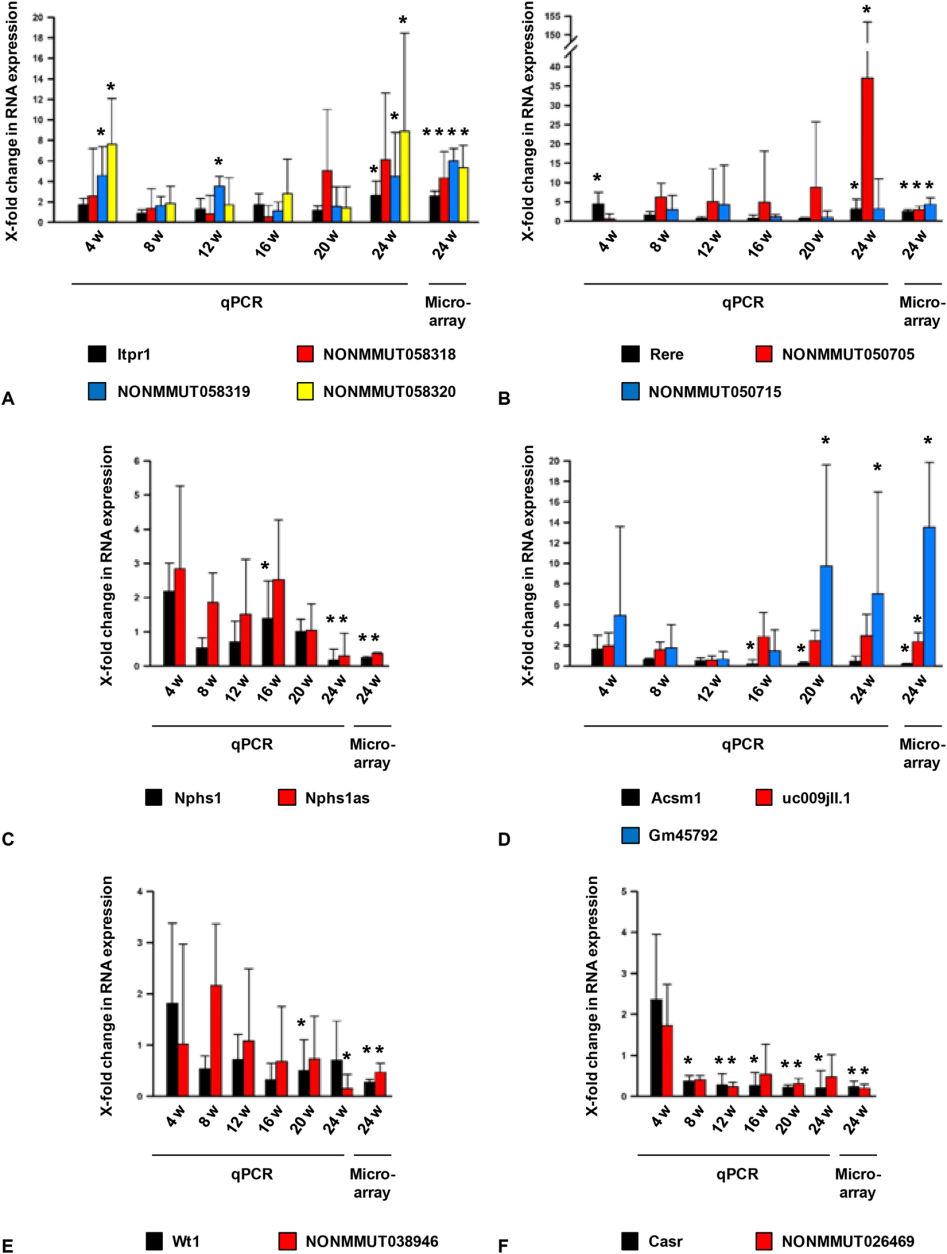
Validation of glomerular gene expression by qPCR in the course of time in BTBR WT and BTBR ob/ob mice and the result of the microarray for 24 weeks old mice (for selected genes). The qPCR analysis was performed for the following ages: four, eight, twelve, 16, 20, and 24 weeks (4 w, 8 w, 12 w, 16 w, 20 w, 24 w), also presented the result of the microarray analysis for 24 weeks old mice (24w). For qPCR and microarray analyses, we used RNA extracted from sieved glomeruli from 3–6 animals (details see below) as independent samples. (**A**) *Itpr1* and its sense lncRNAs *NONMMUT058318*, *NONMMUT058319*, and *NONMMUT058320*. (**B**) *Rere* and its sense lncRNAs *NONMMUT050705* and *NONMMUT050715*. (**C**) *Nphs1* and its antisense lncRNA *Nphs1as*. (**D**) *Acsm1* and its antisense lncRNAs *uc009jll*.*1* and *Gm45792*. (**E**) *Wt1* and its neighboring lncRNA *NONMMUT038946*. (F) *Casr* and its neighboring lncRNA *NONMMUT026469*. Black bars represent the fold change of the protein-coding gene for age-matched murine genotypes. Red, blue or yellow bars correspond to the associated sense, antisense, or neighboring lncRNA for age-matched murine genotypes. Error bars indicate the standard deviation of the fold change. For qPCR experiments: The expression was normalized to *cyclophilin B*. Statistical analysis was conducted using one-way ANOVA. *p < 0.05 comparing age-matched groups; n = 4–6; For microarray analysis: The expression was normalized to internal controls (default setting). Statistical analysis was based on the ANOVA method ebayes. FDR *p < 0.05 comparing 24 weeks old BTBR WT and ob/ob mice; n = 3–4.

Within the transcription unit of inositol 1,4,5-trisphosphate receptor type 1 (*Itpr1*), six sense lncRNAs in total are encoded. Three of them were differentially expressed in our array: *NONMMUT058318*, *NONMMUT058319*, and *NONMMUT058320*. In our qPCR experiment, we could ascertain significant expression changes for *Itpr1*, *NONMMUT058319*, and *NONMMUT058320* (Fig. 3A). The p-value of *NONMMUT058318* was just above the significance level (p = 0.05). Arginine glutamic acid dipeptide (RE) repeats (*Rere*) overlaps with six lncRNAs in sense direction, too. Of these, *NONMMUT050705* and *NONMMUT050715* exhibited a differential expression pattern (Supplementary Table 2). Here, the differential expression could be confirmed by qPCR for *Rere* and its sense lncRNA *NONMMUT050705* (Fig. 3B).

As antisense lncRNA-mRNA-pairs, we selected Nephrin (*Nphs1*) and its antisense lncRNA *Nphs1as*. Both transcripts were differentially expressed in the microarray and the qPCR analysis (Fig. 3C). *Uc009jll*.*1* and *Gm45792* represent antisense transcripts to the acyl-CoA synthetase medium chain family member 1 (*Acsm1*) (Supplementary Table 3). For this pairing, the differential expression could be validated only for *Gm45792* (Fig. 3D).

Wilms tumor-1 (*Wt1*) and calcium-sensing receptor (*Casr*) were differentially co-expressed with their neighboring lincRNAs *NONMMUT038946* and *NONMMUT026469*, respectively (Supplementary Table 4). By qPCR, the differential expression could be confirmed only for *NONMMUT038946* (Fig. 3E) and *Casr* (Fig. 3F). However, *Wt1* was differentially expressed in 20 weeks old mice and *NONMMUT026469* had a p-value only slightly above the significance level (p = 0.07).

### Cell-specific expression of lncRNA-mRNA-pairs

**Figure 4:**
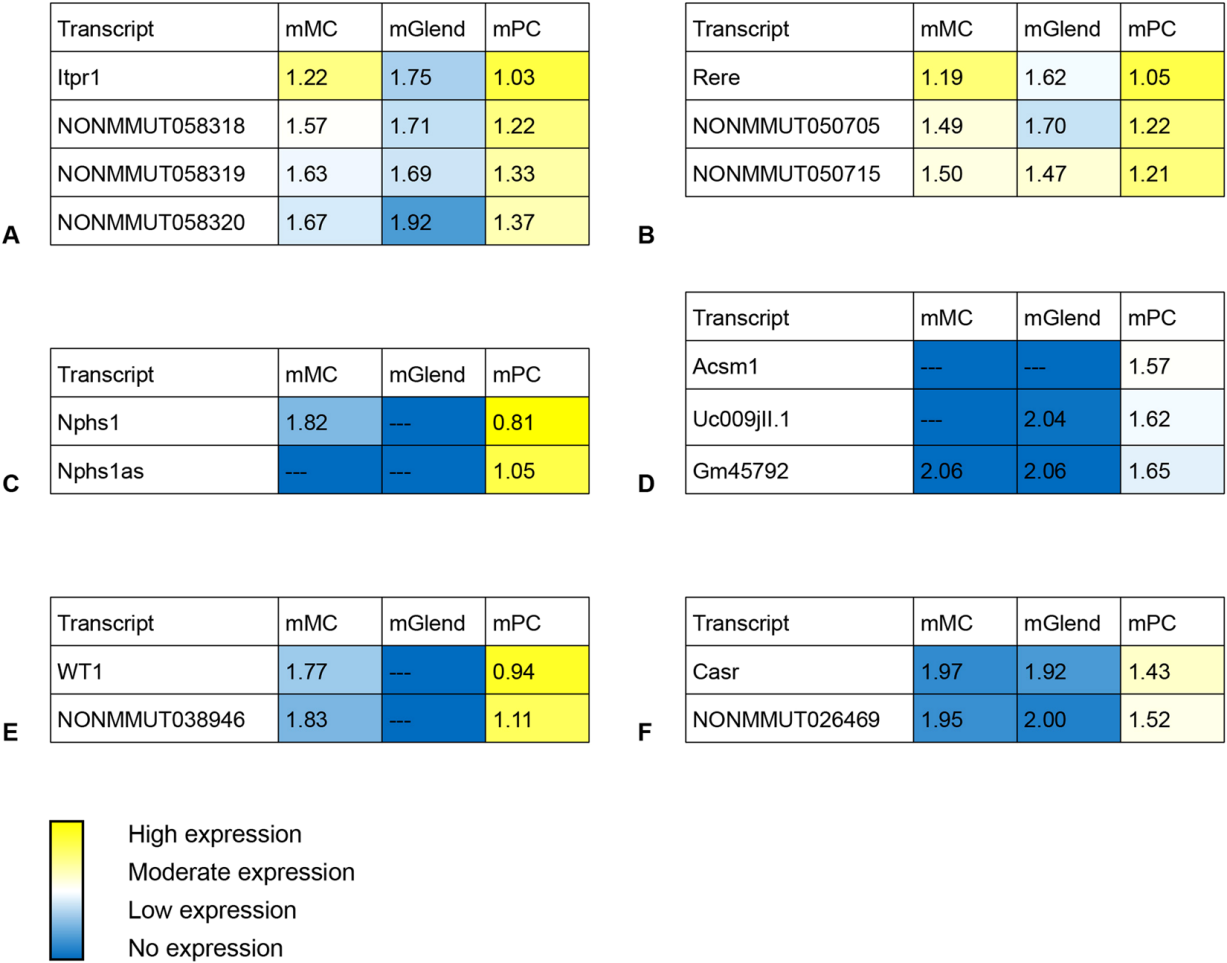
Localization of glomerular expressed transcripts using RNA from murine mesangial cells (mMC), murine glomerular endothelial cells (mGlend), and murine podocytes (mPC). (**A**) *Itpr1* and its sense lncRNAs *NONMMUT058318*, *NONMMUT058319*, and N*ONMMUT058320*. (**B**) *Rere* and its sense lncRNAs *NONMMUT050705* and *NONMMUT050715*. (**C**) *Nphs1* and its antisense lncRNA *Nphs1as*. (**D**) *Acsm1* and its antisense lncRNAs *uc009jll*.*1* and *Gm45792*. (**E**) *Wt1* and its neighboring lncRNA *NONMMUT038946*. (**F**) *Casr* and its neighboring lncRNA *NONMMUT026469*. Values represent the ratio of the C_T_ of the transcript vs. C_T_ of *cyclophilin B*. A low ratio (<1.0) indicates a high expression and is highlighted in yellow, while a high ratio (>1.8) indicates a low expression and is highlighted in blue.—portends that no expression was detected.

Next, we aimed to specify the cell type(s), which expressed the lncRNA-mRNA-pairs described above. The following qPCR experiment was based on RNA from a murine mesangial cell line (mMC), a glomerular endothelial cell line (mGLEND), and primary podocytes from BTBR WT mice. To show relative expression levels, the C_T_ value of the selected gene was normalized to the C_T_ value of peptidylpropyl isomerase B (cyclophilin B), receiving rations between 0.8 and 2.06 (Fig. 4A–F). A low ratio reflects a high expression. In most cases the expression of the mRNA was higher than the related lncRNA(s).

*Itpr1* (Fig. 4A), *Rere* (Fig. 4B), and their adjacent lncRNAs were expressed in all cell types whereby both mRNAs and their lncRNA exhibited the lowest expression in mGLENDs. *Nphs1* (Fig. 4C) and *Wt1* (Fig. 4E) represent podocyte markers. Together with their antisense or neighboring lncRNA, respectively, they were highly expressed in podocytes. As expected, these transcripts displayed either a very low or no expression in mMCs and mGLENDs. Comparably to this, we detected moderate levels of *Acsm1* (Fig. 4D), *Casr* (Fig. 4F) and their related lncRNAs in podocytes while mGLENDs or mMCs showed either a very low or no expression. In summary, these data provide strong evidence for a cell-specific lncRNA expression in the development of experimental diabetic nephropathy.

### Functional enrichment analysis

For functional enrichment analysis of coding genes associated with differentially expressed lncRNAs, we used the Database for Annotation, Visualization and Integrated Discovery (DAVID) for gene ontology (GO) biological processes. For pathway enrichment analyses, the Kyoto Encyclopedia of Genes and Genomes (KEGG) was deployed. We found 15 significantly enriched GO biological processes for genes assigned to intragenic lncRNAs (Table 2), but only one for genes assigned to lincRNAs (“liver development”). GO biological processes for genes assigned to intragenic lncRNAs were mainly related to four cellular processes: organization of cellular architecture (“cell morphogenesis”; “actin filament organization”; 26 genes), ubiquitination (“protein ubiquitination”; “proteasome-mediated ubiquitin-dependent protein catabolic process”; “protein K48-linked ubiquitination”; 71 genes), transport (“transport”; “vesicle-mediated transport”; “intracellular protein transport”; 169 genes), as well as transcription, and transcription control (“transcription (DNA-templated)”; “positive” or “negative regulation of transcription from RNA polymerase II promoter”; “covalent chromatin modification”; 264 genes).

**Table 2:** Significantly enriched GO biological processes for protein coding genes overlapping differentially expressed lncRNAs in BTBR ob/ob mice.

Transport (116 genes)	FDR p value: 5.9*10^−3^
Abcb1b, Abcd3, Abcg2, Agap1, Ano6, Ap1m2, Ap3b1, Ap3s1, Arcn1, Arfgap3, Arfgef2, Atp1a1, Atp1b1, Atp2a2, Atp5c1, Atp6v0a1, Atp6v0a4, Atp6v0d2, Atp6v1b2, Atp6v1h, Cachd1, Cadps2, Chmp3, Cmah, Cnnm2, Col4a3bp, Copb2, Copg2, Cpt1a, Cul3, Cyb5b, Dab2, Dennd1a, Dennd1b, Ergic1, Etfa, Exoc4, Frrs1, Fyttd1, Gcc2, Gria3, Hdlbp, Igf2bp2, Igf2bp3, Igf2r, Itpr1, Kcnb1, Kcnj15, Kcnq1, Kif16b, Lrrc8d, Mpc1, Mpc2, Mtx2, Myo5b, Ndufs4, Ndufv2, Nipal4, Orc4, Osbpl11, Osbpl6, Osbpl8, Osbpl9, Pex7, Pitpnc1, Pkd2, Rab11fip3, Rab1a, Rab22a, Rab5a, Rab7, Rbp2, Rtn3, Scfd1, Scp2, Sdhc, Sec22a, Sec61a2, Sil1, Slc16a1, Slc16a11, Slc16a12, Slc16a2, Slc17a5, Slc20a2, Slc25a10, Slc25a13, Slc25a21, Slc25a26, Slc25a36, Slc29a3, Slc45a4, Slc4a4, Slc8a1, Slc9a3, Slco3a1, Smad3, Snx13, Snx18, Snx9, Sorbs1, Stx16, Stxbp3, Tcte3, Thoc2, Tmed8, Tnpo1, Tom1l1, Tom1l2, Tpcn1, Trappc3, Trim3, Vps13b, Vps54, Vti1a, Xpo7	
**Transcription, DNA-templated (113 genes)**	FDR p value: 4.0*10 ^**−2**^
Adnp, Arid1a, Arid4b, Arid5b, Ascc1, Ash1l, Bcas3, Bcl11b, Bnc2, Brms1, Ccnh, Chchd3, Chd2, Clock, Cnot4, Crebbp, Dido1, Ell2, Elp4, Ep300, Epc1, Erbb4, Esrrg, Fam208a, Fhit, Fli1, Foxp1, Glis3, Gtf2i, Hdac8, Hlf, Hsf2, Ino80d, Jmjd1c, Kat6a, Kdm2a, Kmt2a, L3mbtl2, Lin52, Mapk14, Mbtd1, Med13, Med26, Med27, Mitf, Mkl2, Mxi1, Mycbp2, Ncoa2, Ncoa3, Ncoa5, Ncor1, Nfe2l2, Nfia, Nfyc, Npat, Nr6a1, Nsd1, Park2, Pawr, Pbrm1, Phf5a, Ppara, Ppargc1a, Ppargc1b, Prdm10, Prkaa2, Pspc1, Rb1, Rbpms, Rere, Rora, Rreb1, Setd2, Sfmbt1, Sirt1, Ski, Sltm, Smad3, Smarcc2, Sox30, Ssbp3, Tada2a, Taf1, Tbl1x, Tbl1xr1, Tcf20, Tcf25, Tead1, Tef, Tfap2b, Tfdp2, Thrb, Trim24, Trps1, Tsc22d1, Tshz2, Txlng, Vdr, Wasl, Whsc1l1, Wwc1, Zbtb20, Zbtb5, Zfp280d, Zfp638, Zfp710, Zfp827, Zhx1, Zhx2, Zhx3, Zmiz1, Zmynd11	
**Positive regulation of transcription from RNA polymerase II promoter (67 genes)**	FDR p value: 5.0*10 ^**−2**^
App, Arid4b, Ash1l, Auts2, Bcas3, Bcl11b, Bmp6, Ccnh, Clock, Crebbp, Cys1, Dot1l, Egfr, Ep300, Epc1, Esrrg, Fgfr2, Fhod1, Fli1, Foxp1, Glis3, Hexb, Hsf2, Kmt2a, Lrp6, Map2k5, Mapk14, Med13, Mitf, Mkl2, Mllt10, Ncoa2, Ncoa3, Nfe2l2, Nfia, Nfyc, Nipbl, Nr6a1, Nrg1, Park2, Pkd2, Ppara, Ppargc1a, Ppargc1b, Ppp1r12a, Ppp3ca, Prpf6, Rb1, Rhoq, Rora, Sirt1, Ski, Smad3, Ssbp3, Taf1, Tbl1x, Tbl1xr1, Tcf20, Tead1, Tef, Tfap2b, Thrb, Ube3a, Vdr, Wwox, Yes1, Zmiz1	
**Negative regulation of transcription from RNA polymerase II promoter (55 genes)**	FDR p value: 8.5*10 ^**−3**^
Arid1a, Arid5b, Bmp6, Brms1, Ccnd3, Chchd3, Cpeb3, Crebbp, Cul3, Dlg1, Ep300, Epc1, Fgf9, Fgfr2, Fnip1, Fnip2, Foxp1, Glis3, Map2k5, Mdm4, Mitf, Mxi1, Ncoa2, Ncor1, Nfia, Nipbl, Nr6a1, Nsd1, Park2, Pawr, Pcbp3, Ppara, Ptpn2, Rb1, Rreb1, Sirt1, Ski, Smad3, Smarcc2, Tbl1x, Tbl1xr1, Tcf25, Tfap2b, Thrb, Trps1, Usp3, Vdr, Wwc1, Wwc2, Zbtb16, Zbtb20, Zhx1, Zhx2, Zhx3, Zmynd11	
**Protein ubiquitination (39 genes)**	FDR p value: 9.3*10 ^**−4**^
Arih1, Cul3, Dcaf5, Dcaf8, Dtx4, Enc1, Fbxo11, Fbxo18, Fbxo21, Fbxw7, Itch, Kcmf1, Klhl24, Klhl3, Med27, Mid2, Mycbp2, Nfe2l2, Nsmce2, Park2, Pdzrn3, Peli2, Rbx1, Rnf111, Rnf13, Rnf169, Rnf38, Sh3rf1, Sirt1, Spsb4, Trim24, Trim3, Ubac1, Ube2b, Ube3a, Ube4a, Ube4b, Wwp1, Znrf3	
**Covalent chromatin modification (29 genes)**	FDR p value: 5.3*10 ^**−3**^
Arid1a, Ash1l, Chd2, Epc1, Hdac8, Jmjd1c, Kansl1, Kansl3, Kat6a, Kdm2a, Kdm6a, Kmt2a, L3mbtl2, Mbtd1, Ncor1, Nsd1, Pbrm1, Prkaa2, Rb1, Setd2, Sfmbt1, Smarcc2, Tbl1xr1, Tdrd3, Tlk1, Tlk2, Usp3, Whsc1l1, Zmynd11	
**Vesicle-mediated transport (28 genes)**	FDR p value: 6.4*10 ^**−4**^
Ap1m2, Ap3b1, Ap3s1, Arcn1, Arfgap3, Arfgef2, Bcas3, Clint1, Copb2, Copg2, Cul3, Ergic1, Fnbp1l, Myo5b, Picalm, Rab11fip3, Rab1a, Rtn3, Scfd1, Sec. 22a, Spag9, Stx16, Stx7, Stxbp3, Stxbp6, Tbc1d4, Trappc3, Vti1a	
**Intracellular protein transport (25 genes)**	FDR p value: 2.0*10 ^**−2**^
Ap1m2, Ap3b1, Ap3s1, Copb2, Copg2, Evi5, Klc1, Rab1a, Rab7, Rabgap1, Rabgap1l, Rffl, Sec. 24b, Snx13, Snx18, Snx9, Stx16, Stx7, Tbc1d4, Tlk1, Tnpo1, Tom1l1, Tom1l2, Vti1a, Xpo7	
**Endocytosis (21 genes)**	FDR p value: 2.9*10 ^**−2**^
Aak1, App, Atp6v1h, Clint1, Csnk1g3, Dab2, Dennd1a, Fcho2, Fnbp1l, Lrp1b, Lrp6, Lrrk2, Pacsin2, Picalm, Rab1a, Rab22a, Rab5a, Sgip1, Snx18, Snx9, Synj2bp	
**Proteasome-mediated ubiquitin-dependent protein catabolic process (20 genes)**	FDR p value: 8.5*10 ^**−3**^
Abtb2, Clock, Cul3, Edem3, Faf1, Nfe2l2, Park2, Psmd1, Rbx1, Rffl, Rnf111, Rnf216, Rnf38, Sirt1, Tbl1x, Tbl1xr1, Ube2b, Ube2w, Ube4b, Wwp1	
**Protein dephosphorylation (20 genes)**	FDR p value: 4.9*10 ^**−3**^
Cdc14b, Mtmr3, Nceh1, Ppm1b, Ppm1h, Ppp1cb, Ppp1cc, Ppp1r12a, Ppp3ca, Pptc7, Ptp4a2, Ptpn11, Ptpn18, Ptpn2, Ptpn3, Ptpn4, Ptprf, Ptprj, Smek2, Ssh2	
**Cell morphogenesis (13 genes)**	FDR p value: 4.3*10 ^**−2**^
Add1, Col4a3bp, Cul3, Egfr, Lipa, Map7, Mapk14, Nrg1, Rhoa, Scfd1, Shroom4, Thoc2, Vdr	
**Actin filament organization (13 genes)**	FDR p value: 4.6*10 ^**−2**^
Akap2, Arhgap6, Diaph1, Diaph2, Dlg1, Pakap, Ppargc1b, Ptk2b, Shroom4, Sirpa, Sorbs1, Sorbs2, Wasl	
**Protein K48-linked ubiquitination (12 genes)**	FDR p value: 4.3*10 ^**−3**^
Itch, Klhl3, March6, Park2, Rffl, Rnf216, Ube2b, Ube2e2, Ube2e3, Ube2g1, Ube2k, Ube3a	
**Circadian regulation of gene expression (11 genes)**	FDR p value: 4.7*10 ^**−2**^
Clock, Kmt2a, Ncoa2, Ncor1, Ppara, Ppargc1a, Ppp1cb, Ppp1cc, Rbm4b, Rora, Sirt1	

Significantly enriched GO biological processes of genes being associated with a differentially expressed intragenic lncRNA (sense or antisense) in the glomeruli of eight, 16, and 24 weeks old BTBR ob/ob mice detected by DAVID with FDR corrected p-value, sorted descending by involved genes per biological process.

Regarding the KEGG analysis, eight pathways, including 121 genes, were significantly enriched for genes associated to intragenic lncRNAs (Table 3). Most of these pathways play an important role in the development and progression of diabetes, e. g.: “glucagon signaling”, “protein processing in endoplasmic reticulum”, “ubiquitin mediated proteolysis”, or “insulin resistance”. For neighboring lncRNAs, we detected an enrichment of genes being involved in the pathways “systemic lupus erythematosus” and “biosynthesis of unsaturated fatty acids”. In conclusion, coding genes, associated with differentially expressed lncRNAs, were involved in different GO biological processes and KEGG pathways than the DEGs, previously described^25^.

**Table 3:** Significantly enriched KEGG pathways for protein coding genes overlapping differentially expressed lncRNAs in BTBR ob/ob mice.

**Regulation of actin cytoskeleton (21 genes)**	FDR p value: 4.8*10 ^**−2**^
Arhgef12, Baiap2, Chrm3, Cyfip2, Diaph1, Diaph2, Egfr, Fgf9, Fgfr2, Gng12, Itgb2l, Itgb6, Ppp1cb, Ppp1cc, Ppp1r12a, Ppp1r12b, Pxn, Rhoa, Ssh2, Vav3, Wasl	
**Protein processing in endoplasmic reticulum (19 genes)**	FDR p value: 3.0*10 ^**−2**^
Canx, Edem3, Man1a, Map3k5, March6, Nfe2l2, Ngly1, P4hb, Park2, Rbx1, Sec24b, Sec61a2, Sil1, Stt3b, Ube2e2, Ube2e3, Ube2g1, Ube4b, Ufd1l	
**Ubiquitin mediated proteolysis (17 genes)**	FDR p value: 2.8*10 ^**−2**^
Cul3, Fbxw7, Huwe1, Itch, Park2, Rbx1, Trip12, Ube2b, Ube2e2, Ube2e3, Ube2g1, Ube2k, Ube2w, Ube3a, Ube4a, Ube4b, Wwp1	
**Glucagon signaling pathway (16 genes)**	FDR p value: 8.1*10 ^**−3**^
Camk2b, Cpt1a, Crebbp, Ep300, Itpr1, Ldhb, Phka1, Ppara, Ppargc1a, Ppp3ca, Prkaa2, Prkaca, Prkag2, Prkag3, Sirt1, Smek2	
**Insulin resistance (14 genes)**	FDR p value: 4.8*10 ^**−2**^
Cpt1a, Mgea5, Ppara, Ppargc1a, Ppargc1b, Ppp1cb, Ppp1cc, Prkaa2, Prkag2, Prkag3, Prkce, Ptpn11, Ptprf, Tbc1d4	
**Thyroid hormone signaling pathway (14 genes)**	FDR p value: 4.6*10 ^**−2**^
Atp1a1, Atp1b1, Crebbp, Ep300, Med13, Med27, Ncoa2, Ncoa3, Ncor1, Pfkfb2, Prkaca, Slc16a2, Tbc1d4, Thrb	
**Adherens junction (13 genes)**	FDR p value: 8.8*10 ^**−3**^
Baiap2, Crebbp, Egfr, Ep300, Igf1r, Pard3, Ptprf, Ptprj, Rhoa, Smad3, Sorbs1, Wasl, Yes1	
**Proximal tubule bicarbonate reclamation (7 genes)**	FDR p value: 2.5*10 ^**−2**^
Atp1a1, Atp1b1, Gls, Glud1, Slc25a10, Slc4a4, Slc9a3	

Significantly enriched KEGG pathways of genes being associated with a differentially expressed intragenic lncRNA (sense or antisense) in the glomeruli of eight, 16, and 24 weeks old BTBR ob/ob mice detected by KEGG with FDR corrected p-value, sorted descending by involved genes per biological process.

### Comparison to other data sets

Subsequently, we analyzed if lncRNAs, detected in our array, were also differentially expressed in three other published data sets, available at Gene Expression Omnibus (GEO): GSE87359^28^, GSE86300^29^, and GSE33744^30^. However, in GSE87359^28^ and GSE86300^29^, only male mice were used, and in GSE33744^30^, the sex was not specified.

For 32 lncRNAs, we found a corresponding transcript covered either by data set GSE87359^28^ or all data sets^28–30^ (Table 4). In all microarrays and mouse models, the lncRNAs *AK192544* and Ttc39a opposite strand RNA 1 (*Ttc39aos1*)^31^ were regulated in the same manner (*AK192544* was downregulated, *Ttc39aos1* was upregulated). *AK192544* is an uncharacterized lincRNA, downstream of fatty acid desaturase 2 (*Fads2*) und upstream of *Fads3*. Both, *Fads2* and *Fads3* were differentially expressed in our microarray (Supplementary Table 4). The expression level of functional intergenic repeating RNA element (*Firre*) was elevated in our microarray and in four other murine diabetes models (data sets GSE87359^28^ and GSE33744^30^, but not in GSE86300^29^). In three DN models, *Nphs1as*, the intronic sense lncRNA *B930095g15rik*, and the lincRNA nuclear paraspeckle assembly transcript 1 (*Neat1*) were regulated in the same manner like in our microarray.

**Table 4:** Comparison of differentially expressed lncRNAs in various murine diabetes models.

Entrez Gene ID	Gene Symbol/Transcript (more than one annotation possible)	Fold change in the present microarray	Fold change in other data sets				
			GSE87359	GSE86300	GSE33744 STZ-DBA	GSE33744 db/db	GSE33744 db/dbeNOS^−/−^
100502670	Gm16010; NONMMUT070579; ENSMUST00000145410; uc009rcz.1	0.16	1.81				
85029	Rpph1; NONMMUT020725; ENSMUST00000175096; uc029six.1	0.22	1.18				
102635415	Halr1; NONMMUT056934; ENSMUST00000155758; linc1547	0.33	1.44				
56473	NONMMUT033869; uc008gpa.1; AK192544	0.36	0.88	0.77	0.56	0.41	0.53
445267	Nphs1as; NONMMUT060585; ENSMUST00000152625; NR_004443	0.37	1.01	0.90	1.04	0.94	0.94
77610	C330002G04Rik; NONMMUT034206; ENSMUST00000181623; KnowTID_00004213	2.01	0.98				
102641860	Gm26578; NONMMUT065121; ENSMUST00000181702	2.03	0.99				
71122	4933408N05Rik; NONMMUT067285; ENSMUST00000139864; NR_045831.1; AC151980.4	2.07	0.93	0.92	1.07	1.25	0.73
102640951	Gm15614; NONMMUT051831; ENSMUST00000155976	2.09	0.98				
71125	4933407G14Rik; NONMMUT006207; uc029qyv.1	2.37	1.00	0.98	0.99	1.01	0.88
414116	D630024D03Rik; NONMMUT009155; ENSMUST00000126265; uc007iio.1	2.38	0.98	0.68	0.61	0.33	0.64
102641980	Gm15860; NONMMUT054429; ENSMUST00000137257	2.38	1.29				
621549	Gm11783; NONMMUT045958; ENSMUST00000145581; AI838599	2.39	0.91				
78229	4930596I21Rik; NONMMUT002969; ENSMUST00000152856; uc007cwa.1	2.41	0.96				
654788	4732440D04Rik; NONMMUT000054; ENSMUST00000159618	2.47	0.85	0.87	0.91	1.23	0.91
102635290	Gm12750; NONMMUT048663; ENSMUST00000154561; Ttc39aos1	2.47	1.22	2.32	1.45	3.21	2.38
100662	D930016D06Rik; NONMMUT053449 ENSMUST00000181418; KnowTID_00005652	2.49	0.85	0.71	1.20	0.90	1.14
320268	B930095G15Rik; NONMMUT022481; ENSMUST00000130256; uc007vab.2	2.50	1.10	1.12	0.87	0.70	1.03
102635494	Gm12576; NONMMUT011153; ENSMUST00000135268	2.55	1.09				
102639837	Gm13840; NONMMUT054068; ENSMUST00000149608	2.71	0.93				
666737	4632427E13Rik; NONMMUT062740; ENSMUST00000182408; uc012fpc.1	2.74	1.33				
69771	1810019D21Rik; NONMMUT067006; ENSMUST00000145343; NR_040346.1	2.83	0.96	0.56	1.12	0.63	1.45
108168796	Gm14102; NONMMUT040041; ENSMUST00000147678	2.83	0.88				
100675	AI480526; NONMMUT054413; ENSMUST00000162697	2.85	1.11	0.59	1.15	0.90	1.08
103012	Firre; NONMMUT072398; ENSMUST00000148530; NR_026976.1	2.98	1.30	0.99	1.21	1.37	1.73
102640720	Gm12121; NONMMUT009241; ENSMUST00000131630; NovelTID_00001122	3.73	1.27				
100503565	Gm9926; NONMMUT032727; KnowTID_00003867; uc029tnj.1	4.15	1.09				
319572	C730027H18Rik; NONMMUT006145; KnowTID_00000979	4.24	1.08	0.60	0.69	0.42	1.08
100038525	Gm10804; NONMMUT038657; ENSMUST00000167654; uc008lga.2	4.42	0.91	0.74	1.99	0.64	1.09
68400	0610043K17Rik; NONMMUT048348; ENSMUST00000142581; NR_040640; uc008tvm.1	5.48	1.11				
66961	Neat1; NONMMUT033636; ENSMUST00000185071; KnowTID_00004191	6.77	1.36	0.80	1.06	0.97	1.71
100038479	Gm10787; NONMMUT006277	11.04	1.40				

Differentially expressed lncRNAs (column 2) with their Entrez Gene ID (column 1) and their fold change (column 3) in our microarray compared to the fold changes in other murine diabetes models. These were based on the following conditions. Data set GSE87359: whole-kidney RNA extracts from 22 weeks old T2DM db/db mice. Data set GSE86300: glomerular-deprived kidney cortex from 24 weeks old db/db C57BLKS. Data set GSE33744: glomerular RNA from either 22 weeks old streptozotocin-treated DBA/2 mice (STZ-DBA), 24 weeks old db/db C57BLKS (db/db), or 20 weeks old eNOS-deficient db/db C57BLKS mice (db/db eNOS^−/−^). All fold changes were significantly changed in the corresponding array.

## Discussion

LncRNAs represent novel key players in diabetes and DN. To explore the underlying mechanisms of T2DM, we used mice with the BTBR background. These serve as excellent model for DN since BTBR ob/ob animals develop a DN that closely resembles the human phenotype. The development of the DN occurs without further drug administration, which could have serious side effects^24^. Contrarily, most other studies, dealing with lncRNAs in DN, based on cell culture models^16^, applied a T1DM mouse model mediated by the administration of streptozotocin^18^, or used animal models, which failed to develop a reliable renal phenotype^32,33^. With our comprehensive study, we show for the first time expression changes of lncRNAs, specifically in glomerular tissue, using the very reliable BTBR mouse model for T2DM.

In 24 weeks old BTBR ob/ob mice, we identified the largest number of both, DEGs (as shown previously^25^) and differentially expressed lncRNAs. But while approx. 70% of all differentially expressed mRNAs were downregulated^25^, more than 90% of all differentially expressed lncRNAs were upregulated. Here, intronic sense lncRNAs represented the largest group. Others, studying the murine model of obstructive nephropathy^14^, made comparable observations. Reasons for this phenomenon need to be elucidated. However, epigenetic mechanisms could play a role - either based on differences in methylation patterns between mRNA and lncRNA promoters (and transcriptional units) or based on changes in the methylation pattern as consequence of T2DM. Subsequent studies are necessary to fully understand the underlying mechanisms.

In a chromatin immunoprecipitation DNA sequencing (ChIP seq) experiment using seven human cell types, Melé *et al*. revealed that active promoters of mRNAs and lncRNAs exhibited different histone marks. Promoters of lncRNAs were markedly enriched in H3K9me3 while other marks were characteristic for promoter regions of coding genes^34^. Rodriguez-Rodero *et al*. showed that a T2DM can lead to alterations in the methylation profile (especially hypomethylation) within the gene body^35^. Hypomethylated intron regions can give rise to alternative intragenic transcripts^36,37^. Nevertheless, the methylation pattern might not explain all changes in expression nor the different regulation of mRNAs and lncRNAs, respectively. Regarding the latter, data provided by others suggest that the regulatory regions of coding and non-coding genes harbor distinct transcription factor binding sites^38,39^.

Regarding our previous study^25^, we identified different GO biological processes and KEGG pathways for genes, which were spatially associated with a differentially expressed lncRNA. Here, many genes referred to the GO biological processes of transcription and transcriptional regulation. Herriges *et al*. analyzed the expression of lncRNAs during lung development. Comparably to us, they identified various lncRNAs, which were encoded close to transcription factor loci. Both exhibited differential expression patterns in a spatiotemporal manner^39^. *Smad3* (Table 2) serves as one example for our microarray. Smad proteins are key players in DN and function as downstream effectors in the transforming growth factor β (TGF-β) signaling pathway^40^. An impaired *Smad3* expression negatively affects the expression of various target genes. In case of the gene neuregulin 1 (*Nrg1*; Table 2), the coding gene itself, its intronic sense lncRNA NONMMUT065117, and the neighboring lincRNA NONMMUT065119 were significantly elevated expressed (Supplementary Table 2 and 4). In various murine diabetes models, Nrg1 exhibited a positive effect on glucose homeostasis^41^ and the development of DN^42^.

Additionally, 26 genes involved in cell architecture (GO biological processes: “actin filament organization” and “cell morphogenesis”) were associated with differentially expressed lncRNAs; e. g. the signal regulatory protein α (SIRPα; Table 2), which can interact with NPHS1^43^. SIRPα was shown to be involved in podocyte structure and function^44^.

Beside GO biological processes, also KEGG pathways were significantly enriched in diabetic mice; e. g. “adherens junction” – a pathway, which also contains *Smad3* or *Igf1r* (Table 3). *Igf1r* represents another diabetes-associated gene, whose locus overlaps with six differentially expressed lncRNAs. Ubiquitin-protein ligases are members in the KEGG pathways “ubiquitin mediated proteolysis” and “protein processing in endoplasmic reticulum”. This further supports the relevance of our data since DN is related to endoplasmic reticulum stress (reviewed by Fan *et al*.)^45^.

These examples underline the relevance of lncRNAs in controlling gene expression. Since lncRNAs can affect not only RNA transcription but also splicing or translation^6,7^, they have a substantial influence on function, activity, or stability of proteins. This is particularly true for upstream effectors like transcription factors or molecules at the beginning of a signaling pathway. So, even a little number of lncRNAs, regulating their adjacent genes, might have significant impact on cellular networks and processes.

Moreover, we compared our microarray results with other data sets. Here, we could identify several lncRNAs, which were also differentially expressed in other studies. Unfortunately, the total number of these lncRNAs was low since the available microarrays, used some years ago, covered much less lncRNAs. Additionally, only very few lncRNAs have been known by that time. To our knowledge, the sole lncRNA in Table 4, that was described in the context of diabetes, is *Neat1*^46,47^.

In some cases, the lncRNAs’ expression profiles of the other data sets vary compared to our data. Several reasons can be mentioned for that. We conducted our array based on glomerular RNA while in the other data sets the whole-kidney^28^ or glomeruli-deprived renal tissue^29^ were used. In these cases, glomerular RNA was underrepresented or even absent, leading to different results. Another effect might be due to gender differences. In this study, female mice have been investigated. For two other data sets, male mice have been used and for the other data set the sex has not been published. Furthermore, the other groups used mainly db/db C57BLKS mice, which exhibit a considerably less pronounced renal phenotype. BTBR ob/ob mice, however, represent an excellent mouse model for T2DM associated DN^24^, which does not require any other treatment, like a special diet or injection of agents as streptozotocin, which could have side effects on gene expression.

The strength of our study is the large number of transcripts being covered by the microarray. This provides an excellent summary of the glomerular transcriptome. We used isolated glomeruli because in whole-kidney samples glomerular RNA is very underrepresented. A limitation of the current study is that we could not provide functional data in this context. Our aim was to analyze glomerular gene expression under healthy and diabetic conditions and to identify candidates for potentially cis-acting lncRNAs. However, we are aware that not all detected lncRNAs will control the neighboring gene, but regulate a gene in trans.

In conclusion, our data uncover for the first time the expression pattern of glomerular expressed coding and non-coding genes in a reliable murine T2DM model in the progression of time. Additionally, we identified various potentially cis-acting lncRNAs being associated with coding genes. Many of these genes were involved in processes and pathways, which are relevant in DN. Since these analyses were conducted with female BTBR mice, we also aim to analyze male BTBR mice to determine sex-specific differences in DN. However, the most challenging goal in future will be to assign functions to all of the recently discovered lncRNAs and to analyze their role in DN.

## Methods

### Mouse model

Mice derived from the strain BTBR served as model for T2DM with BTBR WT mice as control and leptin-deficient BTBR ob/ob mice as diabetic animals. Heterozygous BTBR ob+/− mice (BTBR.Cg-Lepob/WiscJ, stock number: 004824; The Jackson Laboratory (Sacramento, CA, USA)) were used for breeding. All mice were bred and housed under standard conditions with twelve hours day-night cycle and free access to water and chow. All animal methods and procedures were performed in accordance with national regulations for care and use of animals. Organ harvesting was conducted according to § 4 subsection 3 TierSchG (german animal protection law) and in adherence with regulations of the Central Animal Laboratories of the University of Regensburg, which covered the criteria and requirements for all approval procedures. Organ harvesting is not subject to further approval by the ethics committee of the University Hospital, a licensing committee, or another responsible authority.

### Glomeruli isolation and RNA isolation

For microarray analysis, glomeruli were isolated from either three or four female BTBR WT and ob/ob mice (four weeks old mice: n = 3; eight, 16, and 24 weeks old mice: n = 4). For qPCR reaction, we also used female WT and diabetic animals at the age of twelve and 20 weeks (n (all age groups) = 6). After cervical dislocation, kidneys were explanted and passed through a series of stainless steel sieves (150 µm, 100 µm, 70 µm, 50 µm). The purity of collected glomeruli (Supplementary Fig. 2) was controlled by an Axiostar plus microscope (Zeiss, Jena, Germany). Subsequent RNA extraction was conducted using the RNeasy Mini Kit (Qiagen GmbH, Hilden, Germany).

### Microarray preparation and processing, data analysis, and data availability

To analyze the expression levels of lncRNAs, we used the GeneChip Mouse Whole Transcriptome 1.0 ST array® (Thermo Fisher Scientific Inc., Waltham, MA, USA). The technical implementation was conducted at the Center of Excellence for Fluorescent Bioanalytics” (Regensburg, Germany; www.kfb-regensburg.de) as described previously^25^. The data were obtained as CEL files and imported into the Transcriptome Analysis Console (TAC) software 4.0. The TAC software was used to calculate summarized probe set signals (based on the SST-RMA algorithm5), average signal values, and FDR p-values. Probe sets with a fold change above 2.0-fold, or below 0.5-fold, and a FDR p-value lower than 0.05 were considered as significantly regulated. Microarray data were submitted to the GEO repository and are accessible through accession number GSE106841.

### Cell culture experiments and isolation of primary podocytes

Our two cell lines (mMCs and mGLENDs) were cultured under standard conditions at 37 °C, in an atmosphere of 5% CO_2_/95% air. MMCs were cultured in DMEM medium (21855, Life Technologies, Carlsbad, CA) supplemented with 5% fetal calf serum (FCS) (Sigma Aldrich; St. Louis, MO). For mGLENDs, DMEM medium (31966, Life Technologies, Carlsbad, CA), supplemented with 10% FCS (Sigma Aldrich; St. Louis, MO), 5 ml non-essential amino acids solution (100x), 0.01% β-mercaptoethanol, and 40 µl mouse VEGF (PromoCell, Heidelberg, Germany) was used. In both cell culture media, 100 U/ml penicillin, 100 g/ml streptomycin (both Life Technologies, Carlsbad, CA), was added. For RNA extraction, cells were harvested after reaching 80% confluency.

Preparation of primary podocytes was conducted as described previously^48^. Briefly, magnetic beads were applied to isolate glomeruli from explanted murine kidneys. Glomeruli were digested and sheared with a 27-G needle. After controlling the digestion result with a fluorescence microscope, cells were separated using a Mo-Flo cell sorter (Beckman Coulter, Krefeld, Germany) with a laser excitation at 488 nm (Power 200 mW) and a sheath pressure of 60 PSI. RNA extraction was immediately performed after isolation of podocytes^48^.

### qPCR experiments for validation of glomerular and cell-specific expression

To validate the microarray results, qPCR experiments were conducted. We used sieved glomeruli (Supplementary Fig. 2) from six female animals per group (primer sequences: Supplementary Table 5). Analysis of cell-specific expression of lncRNA-mRNA-pairs was based on RNA extracted from mMCs, mGLENDs and primary podocytes. After cDNA synthesis employing M-MLV reverse transcriptase (Promega, Mannheim, Germany), all samples were run in triplicates using the ViiA 7 Real-Time PCR System (Thermo Fisher Scientific Inc., Waltham, MA, USA). The expression was normalized to cyclophilin B. The fold change in expression was calculated using the 2^−ΔΔCT^ method. Statistical analysis was performed using IBM SPSS Statistics Version 21. Normal distribution or homogeneity of variances were ascertained by the Shapiro-Wilk and Levene’s test, respectively, before conducting an analysis of variance (ANOVA). Statistical difference was set at the 5% level of probability.

### Functional enrichment analysis

For functional enrichment analyses, we identified protein-coding genes, whose genomic loci overlapped or neighbored differentially expressed lncRNAs. The aim was to discover enriched processes and pathways, which could be controlled by spatially associated lncRNAs. The Database DAVID 6.8 (https://david.ncifcrf.gov/) was used for gene clustering and detection of GO biological process. KEGG pathway enrichment analyses (http://www.genome.jp/kegg/kegg2.html) were used to identify pathways. A FDR p < 0.05 after correction for multiple testing (Benjamini–Hochberg) was set as a threshold^49,50^.

### Comparison to other data sets

To examine if differentially expressed lncRNAs in our microarray were also differentially expressed in other studies, we investigated three further microarray data sets, which addressed five murine DN models. These data sets were accessible through GEO (https://www.ncbi.nlm.nih.gov/gds/) via accession number GSE87359^28^, GSE86300^29^, and GSE33744^30^. The first data set, GSE87359, based on whole-kidney RNA extracts from 22 weeks old male T2DM db/db mice^28^. In data set GSE86300, glomerular-deprived kidney cortex (kidney proximal tubule) gene expression from 24 weeks old male db/db C57BLKS mice was analyzed^29^. Through data set GSE33744, the glomerular expression profiles of three different mouse models were investigated: 22 weeks old streptozotocin-treated DBA/2 mice (STZ-DBA), 24 weeks old db/db C57BLKS (db/db), and 20 weeks old eNOS-deficient db/db C57BLKS mice (db/db eNOS^−/−^)^30^. In this data set, the sex of the mice was not stated. The raw data were normalized using Robust Multichip Algorithm (RMA) and annotated by Mouse Entrez Gene custom CDF annotation version 22 (http://brainarray.mbni.med.umich.edu/Brainarray/default.asp). To identify differentially expressed genes, the SAM (Significance analysis of Microarrays) method was applied using TiGR (MeV, Version 4.8.1)^51^. A q-value below 5% was considered to be statistically significant. Based on the Entrez Gene ID, we identified equivalent transcripts. Only genes with a significant difference in expression were included, statistical significance was assessed using the FDR p-value.

## Supplementary information

Supplementary data
