# Supplementary material for: Glomerular expression pattern of long non-coding RNAs in the type 2 diabetes mellitus BTBR mouse model
Source: Sci Rep. 2019 Jul 5;9:9765. doi: 10.1038/s41598-019-46180-1 (PMC6611801; doi:10.1038/s41598-019-46180-1)
Supplement: Supplementary file 1 — Supplementary data [file 41598_2019_46180_MOESM1_ESM.pdf]

Glomerular expression pattern of long non-coding RNAs in the type 2 diabetes mellitus BTBR mouse model

Simone Reichelt-Wurm*[1], Tobias Wirtz[1], Dominik Chittka[1], Maja Lindenmeyer[2,3], Robert M. Reichelt[4], Sebastian Beck[1], Panagiotis Politis[5], Aristidis Charonis[6], Markus Kretz[7], Tobias B. Huber[3], Shuya Liu[3], Bernhard Banas[1], Miriam C. Banas[1]

1) Department of Nephrology, University Hospital of Regensburg, Germany
2) Department of Internal Medicine IV - Nephrology, University Hospital of Munich, Germany
3) III. Department of Medicine, University Medical Center Hamburg-Eppendorf, Hamburg, Germany
4) Department of Biochemistry, Genetics and Microbiology, Institute of Microbiology, University of Regensburg, Regensburg, Germany
5) Center for Basic Research, Biomedical Research Foundation of the Academy of Athens, Athens, Greece
6) Center for Clinical, Experimental Surgery and Translational Research, Biomedical Research Foundation of the Academy of Athens, Athens, Greece
7) Institute of Biochemistry, Genetics and Microbiology, University of Regensburg, Regensburg, Germany

Supplementary information

Name	Title	Page
Supplementary Table 1	LncRNAs which were differentially expressed in two or three age groups	2-3
Supplementary Table 2	Differentially expressed genes and their corresponding sense transcripts	4-5
Supplementary Table 3	Differentially expressed genes and their corresponding antisense transcripts	6-7
Supplementary Table 4	Differentially expressed genes and their corresponding neighboring transcripts	8
Supplementary Table 5	Primer sequences of analyzed coding and non-coding genes	9
Supplementary Figure 1	Characterization of BTBR WT and BTBR ob/ob mice in the course of time	10
Supplementary Figure 2	A representative photograph from sieved glomeruli from BTBR mice showing the degree of purity	11
Supplementary Figure 3	Murine karyotype	12
Supplementary Figure 4	Pde4d gene locus with all intragenic encoded lncRNA genes	13

SUPPLEMENTARY DATA

Supplementary Table 1: LncRNAs which were differentially expressed in two or three age groups

Age	Transcript	Regulation	Description	Transcript	Regulation	Description
8 and 16 weeks	NONMMUT026469	↓	LincRNA (Stfa3, Casr)	NONMMUT042033, KnowTID_00004635	↑	LincRNA (w/o)
	NONMMUT065898	↓	Antisense (Lpl)	LOC100861638	↓	Antisense (Lpl)
8 and 24 weeks	NONMMUT008002, KnowTID_00001095	↓	LincRNA (Slc16a7)	NONMMUT042033, KnowTID_00004635	↑	LincRNA (w/o)
	NONMMUT026469	↓	LincRNA (Stfa3, Casr)	NONMMUT067315, KnowTID_00007381	↑	LincRNA (w/o)
	NONMMUT065898	↓	Antisense (Lpl)			
16 and 24 weeks	NONMMUT026469	↓	LincRNA (Stfa3, Casr)	NONMMUT017881	↑	Antisense (Zfp808)
	NONMMUT065898	↓	Antisense (Lpl)	NONMMUT048937	↑	LincRNA (Eri3, Dmap1)
	NONMMUT042033, KnowTID_00004635	↑	LincRNA (w/o)	NONMMUT058096, KnowTID_00006395	↑	LincRNA (w/o)
	NONMMUT070579, ENSMUST0000014541, uc009rcz.1, Gm16010	↓	Antisense (Spsb4)	NONMMUT023016	↑	Sense (Fam173b)<br>LincRNA (Cct5, Cmbl)
	NONMMUT001552	↑	Sense (Prkag3)<br>LincRNA (Ttll4, Cyp27a1, Wnt6, Wnt10a)	uc007hxv.1<br>BC019819	↑	Sense (Camk2b)<br>LincRNA (Ykt6)
	NONMMUT004848, KnowTID_00000887	↓	LincRNA (Nhsl1, Hebp2, Arfgef3)	NONMMUT024412, KnowTID_00002815	↑	LincRNA (A4galt, Arfgap3)
	NONMMUT056934, ENSMUST0000015575, Halr1, linc1547	↓	LincRNA (Hoxa1, Hoxa2)	NONMMUT066116, KnowTID_00007069	↑	LincRNA (Rab8a, Hsh2d, Cib3, Fam32a, Ap1m1)
	NONMMUT031786	↑	LincRNA (Pik3c3)	NONMMUT062715	↓	Antisense (Dlg2)

	NONMMUT016243, KnowTID_00001965	↓	LincRNA (Akr1c6)	NONMMUT008425, KnowTID_00001219	↑	LincRNA (Xbp1, Ccdc117, Ankrd36)
	NONMMUT013364, NovelTID_00001374	↑	LincRNA (Rhob, Slc7a15)	uc029sca.1, A130040M12Rik	↑	LincRNA (w/o)
	NONMMUT070568, KnowTID_00007786	↑	LincRNA (Pxylp1)	NONMMUT033453, KnowTID_00004163		LincRNA (Ndufv1, Gstp1, Gstp2, Cabp2, Cdk2ap2, Aip)
	NONMMUT060091, KnowTID_00006493	↑	LincRNA (w/o)	NONMMUT005529, KnowTID_00000711	↑	LincRNA (w/o)
	NONMMUT016573, KnowTID_00001996	↑	LincRNA (Hist1h2bl, Hist1h2ai, Hist1h3h, Hist1h2bm, Hist1h4j, Hist1h4k, Hist1h2ak, Hist1h2bn, Hist1h1b, Hist1h3i, Hist1h2an, Hist1h2bp, Hist1h2br, Hist1h2ao, Hist1h4m)	NONMMUT056587	↑	LincRNA (Trbv16, Trbv17, Trbv19, Trbv20, Trbv21, Trbv23, Trbv26)
	NONMMUT017501, NovelTID_00001743	↑	LincRNA (Cks2 Secisbp2)	NONMMUT046091, KnowTID_00005242	↑	LincRNA (w/o)
	NONMMUT055891, ENSMUSG00000106874, KnowTID_00006260, Gm20186	↑	LincRNA (Lsm8 Ankrd7)	NONMMUT048938, ENSMUST00000156081, Gm12840	↑	LincRNA (Eri3, Dmap1)
	NONMMUT011314, KnowTID_00001300, A130040M12Rik	↑	LincRNA (Dhx40)	NONMMUT020278, KnowTID_00002271	↑	LincRNA (Dph3, Oxnad1, MsMb, Ncoa4)
8, 16, and 24 weeks	NONMMUT026469	↓	LincRNA (Stfa3, Casr)	NONMMUT042033, KnowTID_00004635	↑	LincRNA (w/o)
	NONMMUT065898	↓	Antisense (Lpl)			

Supplementary Table 1: Column 1 displays the age of mice of BTBR mice; column 2 and 5 show the name(s) of a differentially expressed lncRNA; column 3 and 4 indicate if the lncRNAs is upregulated (↑) or downregulated (↓), column 4 and 6 show the orientation of the lncRNA (antisense or sense) or if it is a intergeninc lncRNA (linc). The coding gene, which is associated with the lncRNA, is enclosed in brackets; lincRNAs without an adjacent coding gene 50 kb up- or downstream are indicated with (w/o)

Supplementary Table 2: Differentially expressed genes and their corresponding sense transcripts

Age	Gene	Fold change	FDR p value	Sense transcript (more than one annotation possible)	Fold change	FDR p value
16 weeks	Prkag3	2.01	$7.3*10^{-3}$	NONMMUT001552	4.23	$1.0*10^{-2}$
24 weeks	Aspa	2.52	$2.6*10^{-2}$	NONMMUT010642	5.40	$1.4*10^{-3}$
	Atxn7l1	2.03	$8.0*10^{-4}$	NONMMUT013897	2.05	$4.3*10^{-2}$
	Ccdc141	4.40	$3.9*10^{-2}$	NONMMUT038073	5.13	$2.0*10^{-2}$
	Cpeb2	2.05	$6,9*10^{-5}$	NONMMUT052099	5.38	$4.0*10^{-4}$
	Cpeb3	2.18	$1.0*10^{-3}$	NONMMUT034599	3.22	$1.0*10^{-3}$
	Cpeb4	2.07	$2.0*10^{-3}$	NONMMUT009161	4.28	$4.6*10^{-3}$
	Cspp1	2.06	$1.8*10^{-2}$	NONMMUT000139	2.35	$3.3*10^{-2}$
	Fam214a	2.49	$3.0*10^{-4}$	NONMMUT070040	2.51	$1.8*10^{-3}$
	Fam214a	2.49	$3.0*10^{-4}$	NONMMUT07004	6.40	$1.0*10^{-4}$
	Fnip2	2.30	$2.6*10^{-2}$	NONMMUT043553<br>ENSMUST00000161319<br>Gm3513	2.86	$1.4*10^{-3}$
	Fnip2	2.30	$2.6*10^{-2}$	NONMMUT043550	3.39	$2.5*10^{-3}$
	Fnip2	2.30	$2.6*10^{-2}$	NONMMUT043551	7.95	$4.0*10^{-2}$
	Frmd4b	3.62	$3.0*10^{-5}$	NONMMUT058138	2.48	$3.0*10^{-4}$
	Frmd4b	3.62	$3.0*10^{-5}$	NONMMUT058137	3.97	$4.3*10^{-3}$
	Frmd4b	3.62	$3.0*10^{-5}$	NONMMUT058134	7.56	$2.7*10^{-2}$
	Gls	2.07	$5.5*10^{-3}$	NONMMUT000877	2.00	$1.5*10^{-3}$
	Gls	2.07	$5.5*10^{-3}$	NONMMUT000875	2.36	$6.7*10^{-3}$
	Gls	2.07	$5.5*10^{-3}$	NONMMUT000869	4.70	$2.8*10^{-2}$
	Gramd1b	5.04	$3,8*10^{-6}$	NONMMUT068680	6.84	$2.4*10^{-3}$
	Gramd1b	5.04	$3,8*10^{-6}$	NONMMUT068679	13.92	$1.1*10^{-2}$
	Gramd1b	5.04	$3,8*10^{-6}$	NONMMUT068681	16.58	$2.3*10^{-3}$
	Gria3	4.14	$2.0*10^{-4}$	NONMMUT072210	11.00	$7.0*10^{-5}$
	Hmgcs2	34.33	$6.0*10^{-4}$	NONMMUT044365	5.39	$1.0*10^{-4}$
	Itpr1	2.56	$1.0*10^{-4}$	NONMMUT058318	4.35	$3.2*10^{-2}$
	Itpr1	2.56	$1.0*10^{-4}$	NONMMUT058320	5.34	$1.0*10^{-4}$
	Itpr1	2.56	$1.0*10^{-4}$	NONMMUT058319	6.01	$1.3*10^{-3}$
	Lrig2	2.06	$2.3*10^{-3}$	NONMMUT044600	4.72	$1.3*10^{-3}$
	Lrig2	2.06	$2.3*10^{-3}$	NONMMUT044599	5.31	$1.5*10^{-3}$
	Lrig2	2.06	$2.3*10^{-3}$	NONMMUT044601	5.54	$6.0*10^{-4}$
	Metap1d	2.00	$3.7*10^{-3}$	NONMMUT037822	2.76	$8.7*10^{-3}$
	Nrg1	2.09	$8.0*10^{-4}$	NONMMUT065117	3.90	$1.0*10^{-4}$
	Pde4c	3.02	$5.0*10^{-4}$	NONMMUT066016	4.49	$1.6*10^{-3}$
	Prkag3	2.44	$1.0*10^{-4}$	NONMMUT001552	5.92	$3.0*10^{-4}$

	Prpf39	2.11	$1.5*10^{-2}$	NONMMUT014398	2.18	0.0160
	Pxmp4	2.04	$1.1*10^{-2}$	NONMMUT040619	8.14	$2.0*10^{-5}$
	Rere	2.53	$8.4*10^{-3}$	NONMMUT050705	3.00	$1.6*10^{-2}$
	Rere	2.53	$8.4*10^{-3}$	NONMMUT050715	4.31	$1.3*10^{-2}$
	Sh3rf1	2.26	$8.2*10^{-3}$	NONMMUT065770	2.17	$1.8*10^{-2}$
	Slco3a1	2.10	$9.0*10^{-4}$	NONMMUT062223	2.00	$1.1*10^{-2}$
	Slco3a1	2.10	$9.0*10^{-4}$	NONMMUT062221	2.68	$2.0*10^{-3}$
	Srcin1	2.11	$3.8*10^{-3}$	NONMMUT011803<br>ENSMUST00000134289<br>Gm11611	2.63	$2.0*10^{-2}$
	Srek1	2.58	$5.6*10^{-3}$	NONMMUT018986	2.15	$1.2*10^{-2}$
	Tacc2	2.47	$2.2*10^{-6}$	NONMMUT063899	2.44	$1.8*10^{-3}$
	Tnrc6a	2.38	$1.1*10^{-2}$	NONMMUT063543	2.86	$3.2*10^{-2}$
	Ttll4	2.17	$1.6*10^{-3}$	NONMMUT001547	2.21	$4.3*10^{-2}$
	Wdr91	2.26	$9.0*10^{-4}$	NONMMUT056333	3.51	$1.7*10^{-3}$
	Whsc1l1	2.18	$3.7*10^{-3}$	NONMMUT064990<br>Gm45343	2.66	$2.6*10^{-2}$
	Zbtb16	8.19	$3.0*10^{-4}$	NONMMUT069015	3.33	$2.1*10^{-2}$
	Cpne3	0.42	$8.4*10^{-3}$	NONMMUT046349	3.20	$11.*10^{-2}$
	Gucy1a2	0.40	$1.5*10^{-2}$	NONMMUT067888	2.67	$3.1*10^{-2}$
	Manea	0.44	$1.4*10^{-2}$	NONMMUT046502	2.87	$3.7*10^{-2}$
	Papss2	0.40	$1.1*10^{-2}$	NONMMUT034475	2.78	$2.4*10^{-2}$
	Prex2	0.29	$2.3*10^{-2}$	NONMMUT000151	2.89	$2.4*10^{-2}$
	Prlr	0.50	$1.6*10^{-2}$	NONMMUT022731	2.09	$1.3*10^{-2}$
	Slc16a12	0.45	$3.4*10^{-3}$	NONMMUT034530	2.62	$4.0*10^{-2}$
	Thsd7a	0.20	$1.2*10^{-3}$	NONMMUT055768	2.01	$2.5*10^{-2}$
	C1qtnf7	0.45	$3.4*10^{-3}$	NONMMUT052109	0.43	$4.4*10^{-2}$
	Magi2	0.37	$8.9*10^{-3}$	NONMMUT051353	0.28	$4.0*10^{-2}$
	Npr3	0.16	$3.8*10^{-3}$	NONMMUT022770	0.49	$2.9*10^{-2}$
	Robo2	0.20	$1.6*10^{-3}$	NONMMUT027254	0.43	$4.4*10^{-2}$

Supplementary Table 2: Column 1 displays the age of mice; column 2 shows differentially expressed protein-coding genes with their x-fold change of expression (BTBR ob/ob vs. WT; column 3), the corresponding FDR p-value (column 4), and the overlapping lncRNA in sense direction (column 5). Column 6 and 7 inform about the lncRNA's x-fold change of expression (BTBR ob/ob vs. WT) and the corresponding FDR p-value. A blue background indicates a downregulation, a yellow background an upregulation.

Supplementary Table 3: Differentially expressed genes and their corresponding antisense transcripts

Age	Gene	Fold change	FDR p value	Antisense transcript (more than one annotation possible)	Fold change	FDR p value
8 weeks	Lpl	0.26	$4.0*10^{-4}$	NONMMUT065898	0.28	$1.0*10^{-3}$
	Lpl	0.26	$4.0*10^{-4}$	LOC100861638	0.35	$7.9*10^{-3}$
	Tmem72	0.26	$2.9*10^{-2}$	NONMMUT058536	0.48	$4.2*10^{-2}$
16 weeks	Ndrg1	2.81	$4.2*10^{-3}$	NONMMUT023810<br>ENSMUST00000170557<br>Gm17035	3.86	$9.6*10^{-3}$
	Lpl	0.22	$9.0*10^{-5}$	LOC100861638	0.24	$1.3*10^{-3}$
	Lpl	0.22	$9.0*10^{-5}$	NONMMUT065898	0.31	$1.3*10^{-3}$
24 weeks	Arrdc3	3.66	$9.1*10^{-5}$	NONMMUT006433<br>ENSMUST00000105760<br>Gm17151	2.54	$3.5*10^{-2}$
	Dot1l	2.45	$6.6*10^{-3}$	NONMMUT018449<br>ENSMUST00000161006<br>1700023H06Rik	2.18	$6.0*10^{-4}$
	Olfr1055	2.13	$2.2*10^{-2}$	NONMMUT038330	4.00	$1.5*10^{-2}$
	Abcg2	0.49	$2.0*10^{-2}$	NONMMUT057170	2.05	$29*10^{-2}$
	Acot3	0.41	$7.3*10^{-3}$	NONMMUT014996	2.82	$1.3*10^{-3}$
	Acsm1	0.25	$1.0*10^{-4}$	NONMMUT063428<br>uc009jll.1<br>uc009jlk.1	2.36	$4.4*10^{-2}$
	Acsm1	0.25	$1.0*10^{-4}$	NONMMUT063430<br>ENSMUST00000207795<br>Gm45792	13.54	$2.6*10^{-3}$
	Cd302	0.44	$4.0*10^{-4}$	NONMMUT037482	2.35	$1.4*10^{-2}$
	Fli1	0.38	$7.9*10^{-3}$	LOC100862152<br>XR_140930.1	3.32	$1.6*10^{-2}$
	Tshz2	0.45	$1.7*10^{-3}$	NONMMUT041414	2.33	$2.3*10^{-2}$
	Lpl	0.19	$5.7*10^{-6}$	LOC100861638	0.13	$5.7*10^{-6}$
	Lpl	0.19	$5.7*10^{-6}$	NONMMUT065898	0.16	$3.8*10^{-6}$
	Nphs1	0.25	$1.1*10^{-2}$	NONMMUT060585<br>ENSMUST00000152625<br>NR_004443<br>Nphs1os	0.37	$9.3*10^{-3}$
	Sept11	0.38	$6.8*10^{-3}$	NONMMUT053138	0.29	$4.2*10^{-3}$

Supplementary Table 3: Column 1 displays the age of mice; column 2 shows differentially expressed protein-coding genes with their x-fold change of expression (BTBR ob/ob vs. WT; column 3), the corresponding FDR p-value (column 4), and the overlapping lncRNA in antisense direction (column 5). Column 6 and 7 inform about the lncRNA's x-fold change of expression (BTBR ob/ob vs. WT) and the corresponding FDR p-value. A blue background indicates a downregulation, a yellow background an upregulation.

Supplementary Table 4: Differentially expressed genes and their corresponding neighboring transcripts

Age (weeks)	Distance (bp)	Gene	Fold change	FDR p value	Strand	Neighboring transcript (more than one annotation possible)	Fold change	FDR p value	Strand	Up-/down-stream
24	147	Fads2	0.38	$4.0*10^{-4}$	-	NONMMUT033869; uc008gpa.1; AK192544	0.36	$4.3*10^{-3}$	-	down
24	366	Pon3	0.31	$4.0*10^{-4}$	-	NONMMUT055603; NovelTID_00004948	0.29	$3.0*10^{-4}$	-	down
24	814	Cyp2j9	2.32	$1.6*10^{-2}$	-	NONMMUT048206	2.34	$1.9*10^{-2}$	-	down
24	1397	Casr	0.17	$1.5*10^{-3}$	-	NONMMUT026469	0.16	$3.0*10^{-4}$	-	down
24	1978	Nrg1	2.09	$8.0*10^{-4}$	-	NONMMUT065119	2.62	$5.0*10^{-3}$	-	up
24	2236	Baat	0.47	$6.4*10^{-3}$	-	NONMMUT047233	0.40	$2.1*10^{-3}$	+	down
24	2462	Frmd4b	3.62	$3.0*10^{-5}$	-	NONMMUT058132; KnowTID_00006398	2.49	$3.8*10^{-3}$	-	down
24	2951	Pde3a	0.39	$4.2*10^{-3}$	+	NONMMUT059325; KnowTID_00006219	0.39	$8.1*10^{-3}$	+	down
8	3687	Slc16a7	0.33	$2.1*10^{-2}$	-	NONMMUT008003; KnowTID_00001096	0.29	$4.8*10^{-2}$	-	down
24	3961	Fads3	0.44	$1.0*10^{-3}$	+	NONMMUT033869; uc008gpa.1; AK192544	0.36	$4.3*10^{-3}$	-	down
24	4142	Top1mt	0.45	$3.1*10^{-2}$	-	NONMMUT023966	0.21	$8.3*10^{-3}$	-	down
24	4637	Itpr1	2.56	$1.0*10^{-4}$	+	NONMMUT058324; KnowTID_00006165	2.07	$2.0*10^{-4}$	+	down
24	4918	Aadac	0.37	$4.6*10^{-3}$	+	NONMMUT043269	4.7	$9.3*10^{-3}$	+	down
24	5629	Acot1	2.47	$2.8*10^{-2}$	+	NONMMUT014993; KnowTID_00001645	2.03	$1.4*10^{-2}$	-	down
24	5702	Slc16a7	0.33	$2.1*10^{-2}$	-	NONMMUT008002; KnowTID_00001095	0.32	$9.1*10^{-3}$	-	down
24	6666	Pde3a	0.39	$4.2*10^{-3}$	+	NONMMUT059326; KnowTID_00006220	0.48	$2.0*10^{-2}$	+	down
24	8587	Cyp2d12	0.45	$9.1*10^{-3}$	+	NONMMUT024377	2.92	$2.4*10^{-2}$	-	up
24	9250	Cox6a2	2.12	$3.7*10^{-2}$	-	NONMMUT063796; KnowTID_00006927	2.38	$3.2*10^{-2}$	-	up
24	11433	Ak4	0.39	$2.7*10^{-2}$	+	NONMMUT048353; ENSMUST00000124786; Gm12798	7.16	$6.0*10^{-4}$	+	up
24	14700	Ak4	0.39	$2.7*10^{-2}$	+	NONMMUT048348; ENSMUST00000142581; NR_040640	5.48	$1.0*10^{-4}$	+	up
24	15943	Nt5c3b	0.46	$5.2*10^{-7}$	-	NONMMUT012011	0.49	$8.2*10^{-3}$	-	up

24	24458	Acot3	0.41	$7.3*10^{-3}$	+	NONMMUT014993; KnowTID_00001645	2.03	$1.4*10^{-2}$	-	up
24	24527	Gstp1	0.42	$4.0*10^{-4}$	-	NONMMUT033453; KnowTID_00004163	2.79	$1.6*10^{-2}$	-	up
24	25119	Prpf38b	2.06	$6.8*10^{-3}$	-	NONMMUT044811; ENSMUST00000141723	4.16	$3.7*10^{-2}$	-	up
24	25415	Mrpl50	0.39	$4.6*10^{-3}$	-	NONMMUT047233	0.40	$2.1*10^{-3}$	+	down
24	25441	Magi2	0.37	$8.9*10^{-3}$	+	NONMMUT051340; KnowTID_00005787	0.48	$2.8*10^{-2}$	-	up
24	25868	Clec14a	0.35	$8.8*10^{-3}$	-	NONMMUT014344	2.87	$3.5*10^{-2}$	+	up
24	26733	Tram1	0.41	$6.0*10^{-4}$	-	NONMMUT000191; KnowTID_00000399	2.02	$4.6*10^{-2}$	-	up
24	28759	Wt1	0.28	$5.1*10^{-3}$	+	NONMMUT038946; KnowTID_00004536	0.47	$4.5*10^{-2}$	-	up
8	30090	Slc16a7	0.33	$2.1*10^{-2}$	-	NONMMUT008011; KnowTID_00001098	0.47	$3.2*10^{-2}$	-	up
24	31485	Id2	0.48	$1.0*10^{-2}$	-	NONMMUT013709	2.72	$2.4*10^{-2}$	+	down
24	34643	Sh3bgrl2	0.41	$1.2*10^{-2}$	+	NONMMUT070284; KnowTID_00007546	2.95	$2.5*10^{-2}$	+	up
24	35143	Alas2	2.38	$1.0*10^{-2}$	+	NONMMUT074311; KnowTID_00008070	2.48	$1.3*10^{-2}$	-	up
24	42355	Lpin1	3.05	$1.*10^{-3}$	-	NONMMUT013502; NovelTID_00001395	2.57	$1.7*10^{-2}$	+	up
24	43736	Plekha7	2.23	$1.6*10^{-3}$	-	NONMMUT063351; Gm44867	2.53	$5.6*10^{-3}$	-	up
24	46393	Cpeb4	2.07	$2.0*10^{-3}$	+	NONMMUT009155; ENSMUST00000126265; D630024D03Rik	2.38	$1.2*10^{-3}$	-	up
24	47668	St3gal6	0.46	$2.9*10^{-3}$	-	NONMMUT027043; KnowTID_00003159	2.58	$3.1*10^{-2}$	+	up
24	48617	Rps13	0.48	$2.0*10^{-4}$	-	NONMMUT063351; Gm44867	2.53	$5.6*10^{-3}$	-	down
24	49917	Tmem150c	0.34	$2.1*10^{-3}$	-	NONMMUT053283	2.25	$8.2*10^{-3}$	+	down

Supplementary Table 4: Column 1 displays the age of mice; column 2 provides information about the shortest distance between a differentially expressed protein-coding gene (column 3) and its neighboring differentially expressed lncRNA (column 7). Column 4 and 8 show the respective x-fold change of expression (BTBR ob/ob vs. WT), column 5 and 9 the FDR p-value, and column 6 and 10 the DNA strand, where the corresponding is gene encoded. Column 11 displays the position of the lncRNA relatively to the neighboring protein-coding gene, with up for an upstream position and down for a downstream position. A blue background indicates a downregulation, a yellow background an upregulation.

Supplementary Table 5: Primer sequences of analyzed coding and non-coding genes

Gene	forward	reverse
Acsm1	5'-CTTGATTCTGCCCCGAGTG-3'	5'-GTCCTTGGCTTTCAGTTGGG-3'
Casr	5'-AGGGCATCATTGAGGGAGAG-3'	5'-CTCGCATCTGTCTCACCACT-3'
Cyclophilin B	5'-TGATCCAGGGTGGAGACTTC-3'	5'-ATTGGTGTCTTTGCCTGCAT-3'
Gm4579	5'-GGCTCCAAAGTCACAACAGG-3'	5'-TGTGTCCAAGGTCTGCTCAC-3'
Itpr1	5'-GCTCAATCCCACCAATGCTG-3'	5'-AGCCACACCTCTTCCTCATC-3'
NONMMUT026469	5'-ACAGCCAGGACTACACAAGG-3'	5'-GCCCTCTGATTTCCATGCAG-3
NONMMUT038946	5'-CACCGTACTCAAGCAAACCC-3'	5'-CAAAAGAGGTCACAGCCCAC-3'
NONMMUT050705	5'-TCTCCCAAGTGCCAGTGTAG-3'	5'-TACAAGTTCCCTTCCGAGCC-3'
NONMMUT050715	5'-TTTGCTGTGCTTACCAGGGA-3'	5'-TCGTGAGTTTGTGTGTTGCA-3'
NONMMUT058318	5'-GTCTGTGTGGCAATCCCTAC-3'	5'-GGCATCTGGTCACGTTCTTC-3'
NONMMUT058319	5'-TAGTTGTCACGGAGGGAAGG-3'	5'-TTGAATGAGAAGGAGCCGAG-3'
NONMMUT058320	5'-TCATGAGTGGAAGGAACAGC-3'	5'-GGGGCTAGAGGAAACAAGTT-3'
Nphs1	5'-TGGATATAGTCTGCACCGTCG-3'	5'-ATCTTCTCCATGTCGTCCAGG-3'
Nphs1as	5'-ACATGAAACTTTCCCATCCCC-3'	5'-GCAGTTTGACATCCCTTGAGG-3'
Rere	5'-TGACACCACTCTCAATGCAC-3'	5'-GCACTTCTCGATCAGCTTGG-3'
Uc009jll.1	5'-GCCTATGGATGTGGGTGTTC-3'	5'-GGCAGAAACAGGCAAGTACC-3'
Wt1	5'-CCAAATGACCTCCCAGCTTG-3'	5'-TGCCCTTCTGTCCATTTCAC-3'

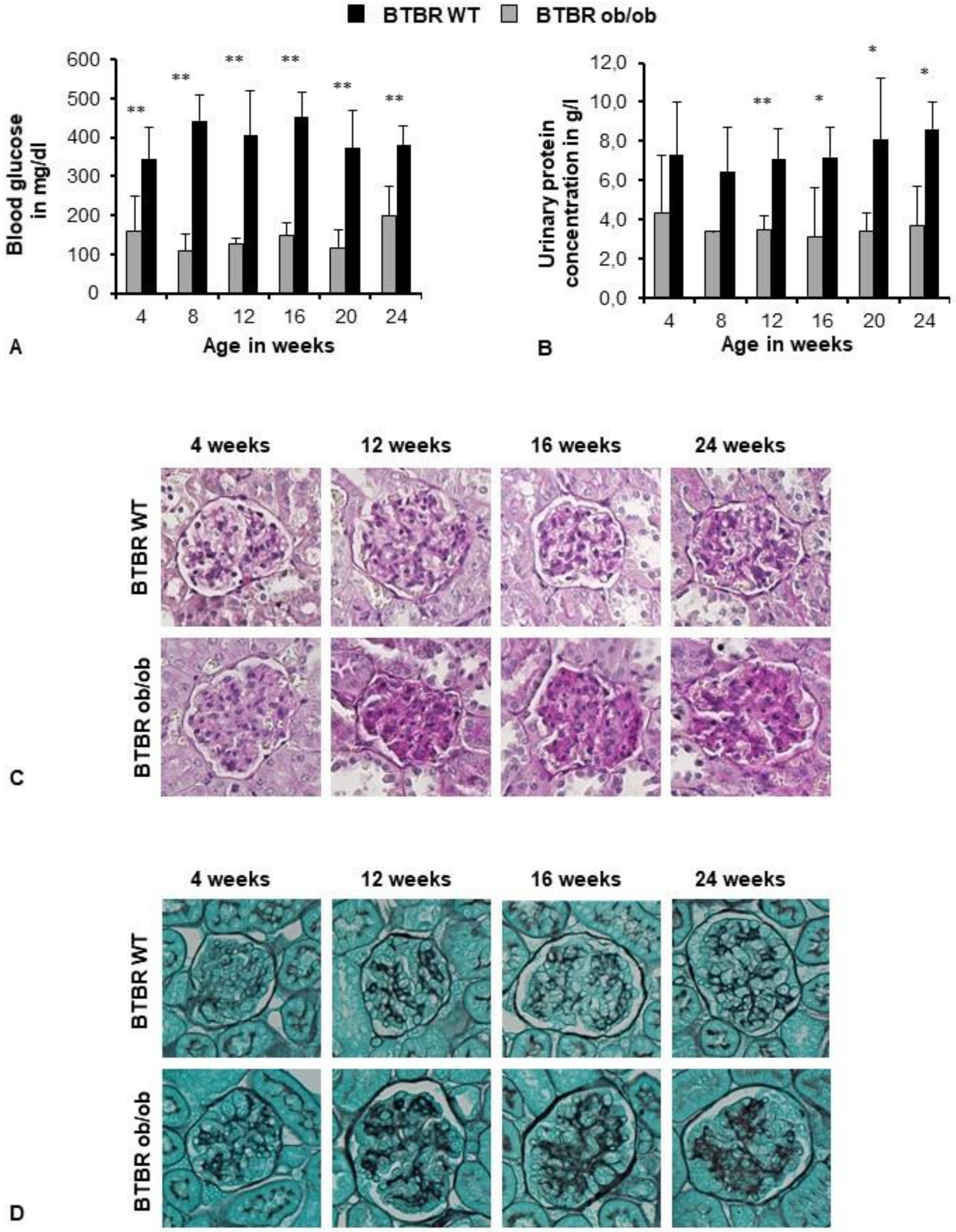

Supplementary Figure 1: Characterization of BTBR WT and BTBR ob/ob mice in the course of time. (A) Blood glucose concentration in mg/dl and (B) urinary protein concentration in g/l in four, eight, twelve, 16, 20 and 24 weeks old BTBR WT and ob/ob mice. Black bars (+SD) represent WT mice, grey bars (+SD) diabetic mice. Statistical analysis was conducted using one-way ANOVA with *) $p < 0.05$; **) $p < 0.01$;. n=6-8. (C) Periodic acid-Schiff stain and (D) silver stain of renal paraffin-embedded sections from four, twelve, 16, 24 weeks old BTBR WT and ob/ob mice. Staining was performed according to standard protocols. Images were taken using a Axiostar plus microscope and AxioVision Release 4.8.1 software (both Zeiss, Jena, Germany). Magnification: 400-fold; n=6-8

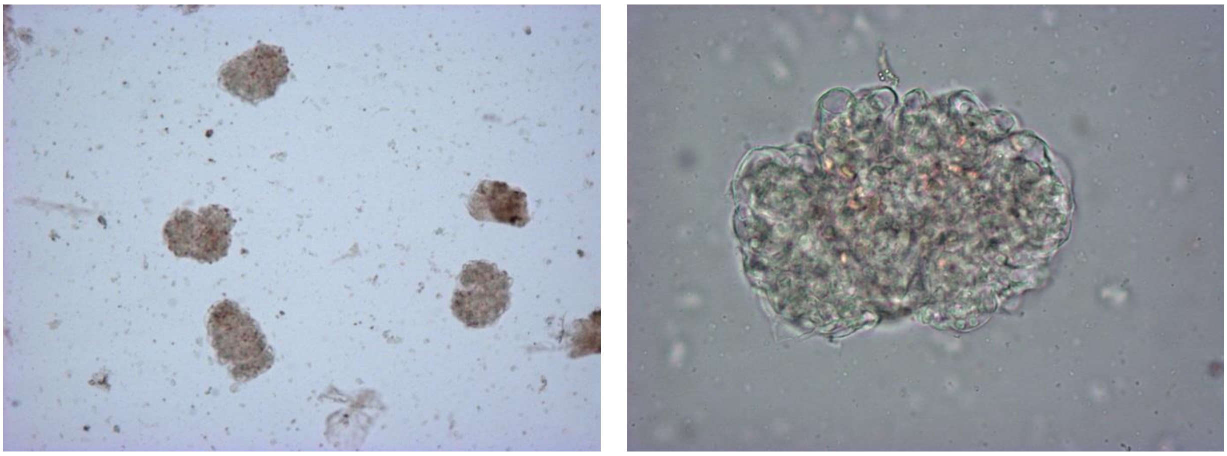

Supplementary Figure 2: A representative photograph from sieved glomeruli from BTBR mice showing the degree of purity. The glomeruli displayed here were collected from 760 µm sieves. Images were taken using a Axiostar plus microscope and AxioVision Release 4.8.1 software (both Zeiss, Jena, Germany).Magnification of 100-fold (left) and 400-fold (right).

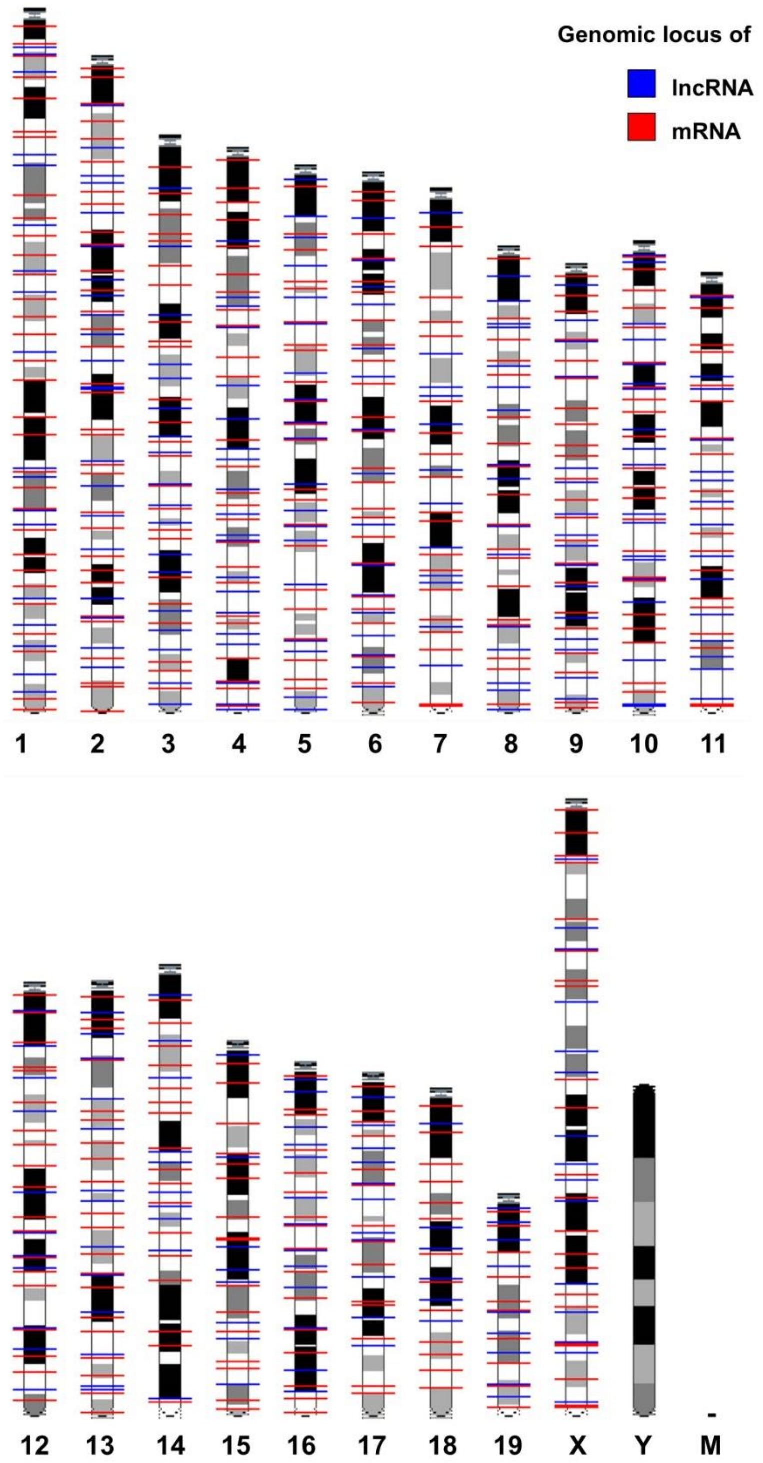

Supplementary Figure 3: Murine karyotype showing the genomic loci of all differentially expressed lncRNAs (blue) and mRNAs (red) encoded on chromosome 1-19, the heterochromosomes X and Y, and the mitochondrial chromosome (M). The karyotype was created using Ensembl Genome Database (www.ensembl.org).

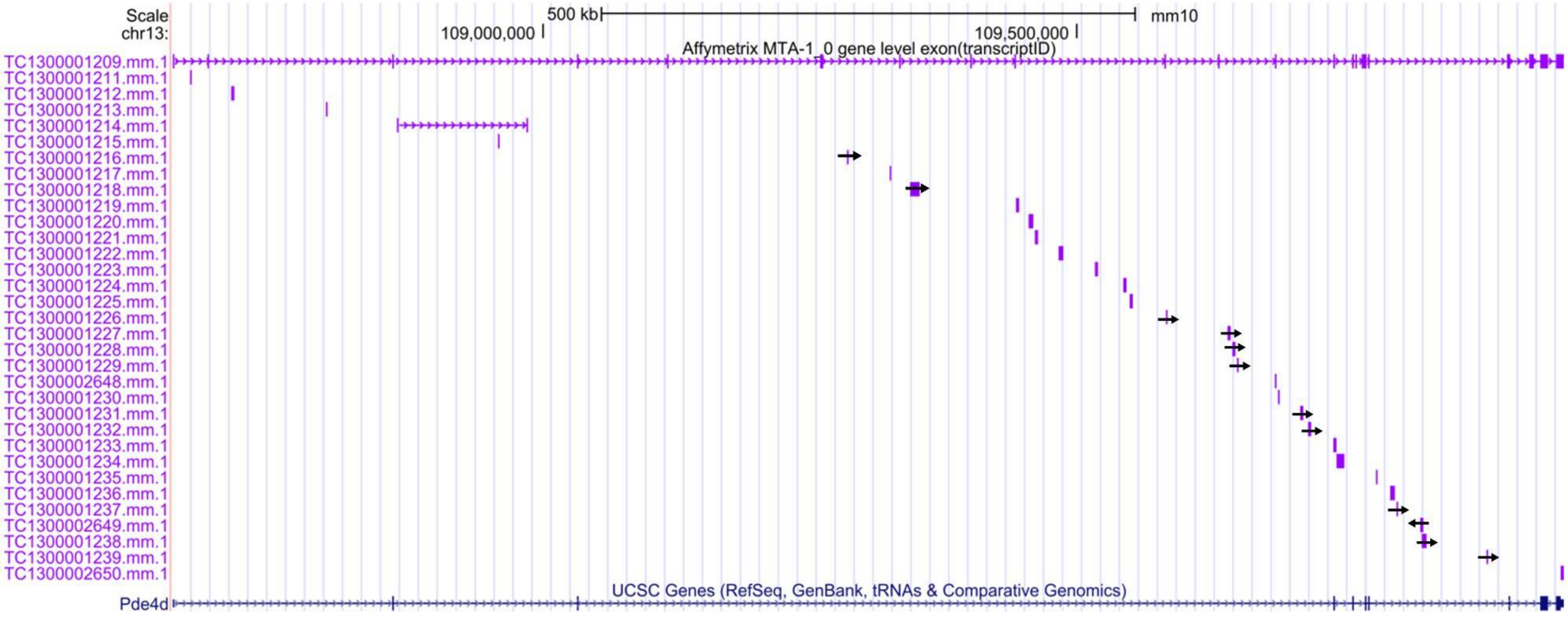

Supplementary Figure 4: Pde4d gene locus with all intragenic encoded lncRNA genes. Pde4d as depicted by the UCSC Genes track on the UCSC Genome Browser (Mouse Assembly mm10) is shown as UCSC gene (blue) and as probe set with the transcript cluster ID TC1000000215.mm (purple). Pde4d is encoded on the + DNA strand. All intragenic lncRNA genes and their transcript cluster IDs which are covered by the array are displayed after loading the Affymetrix MTA-1_0_gene level exon (transcriptID) Track Settings. Additionally, differentially expressed intronic lncRNAs are labeled with an arrow. The direction of the arrow indicates a sense (→) or an antisense (←) orientation.
